# The short-term and long-term effect of burn on organ dysfunction: mechanisms and strategies

**DOI:** 10.3389/fimmu.2026.1933014

**Published:** 2026-10-07

**Authors:** Xuefei Shi, Ying Guo, Rui Lang, Ding Zeng, Xiaohui Ning

**Affiliations:** Department of Burn and Plastic Surgery, The Chinese People’s Liberation Army (PLA) Rocket Force Characteristic Medical Center, Beijing, China

**Keywords:** burns, chronic disease, intervention strategies, multi-organ injury, pathological mechanisms

## Abstract

Burns constitute a form of severe trauma and may affect multi-organ systems. Secondary multi-organ dysfunction is the primary driver of disability, death, and poor prognosis in patients with severe burns, and has become a pressing global public health challenge. In the acute phase of burns, hypovolemic shock and uncontrolled systemic inflammatory response are the most important lethal mechanisms. In addition to localized skin damage, burns can rapidly induce acute dysfunction in the cardiovascular, immune, endocrine, coagulation, musculoskeletal, digestive, and central nervous systems. The pathological pathways of various organs interact and amplify one another in a cascade, forming a self-perpetuating cycle that exacerbates the condition and can rapidly progress to multi-organ dysfunction, leading to premature mortality. This article presents a comprehensive review of burn-related literature published over the past decade. Recent studies have revealed that burns are not merely acute trauma but a chronic disease. Severe burns are characterized by chronic pathological progression. Burn survivors remain in a state of hypermetabolism, chronic inflammation, oxidative stress imbalance, and endocrine disruption following injury, which can lead to organ fibrosis, chronic metabolic abnormalities, and long-term neurocognitive impairment. Their physical motor function, mental and psychological health, and overall quality of life may progressively deteriorate for years or even decades after the injury. This article provides a comprehensive review of the pathological mechanisms underlying acute and chronic multi-organ injury caused by severe burns, outlines comprehensive prevention and treatment strategies combining symptomatic interventions during the acute phase with long-term targeted repair, summarizes the limitations of existing technologies, and explores the application prospects of novel intervention targets and repair techniques, thereby providing a theoretical basis for the standardized prevention and treatment of burn-related multi-organ injury throughout its entire course.

## 1 Introduction

Severe burns are a major public health concern worldwide. There are approximately 180,000 burn-related deaths worldwide each year, the vast majority of which occur in developing countries (1). Causes include thermal sources, chemicals, electricity, and radiation (2). While age-standardized rates have declined, total cases and years lived with disability (YLDs) are rising (3), straining healthcare systems (2, 4), especially in low-and middle-income countries. Overall, children and the elderly are most vulnerable, with common sites being hands, face, and feet (5). However, the epidemiological characteristics of burns vary notably across countries. Elderly African American women and young boys have the highest mortality rates (6, 7). Meanwhile, Chinese researchers found that the incidence of severe burns is higher among children aged 0–4 and adults aged 30–45, with the highest incidence occurring in the summer. Nearly half (48%) of severe burns fall into this category, 77% of which are scalds and flame burns (8). German researchers found that burns occur with clear time patterns, peaking in summer or holidays due to increased activities and fire risks, such as during New Year, highlighting the need for targeted safety interventions (9). Canadian studies show seasonal burn severity varies by age, with most severe burns in summer affecting younger patients (10). Research from India, Palestine, and other regions finds children aged 13–16 have the highest burn incidence (11, 12), with scalds in kitchens as the leading cause (13, 14). Burn-associated mortality fluctuates greatly between geographical zones. India reports a mortality of 14.2% (11), the interval for sub-Saharan Africa runs from 11% to 22.6% (15), while Mexico shows merely 0.6% (16). It is worth noting that the impact of burns is far more than acute injury and immediate death (17). Severe burn survivors often face long-term problems, as hypermetabolic and inflammatory responses can persist for up to three years. This may lead to chronic multi-organ failure, affecting prognosis and causing lifelong physical and psychological challenges (18).

Matched cohort data from Western Australia show burns impact elderly survival beyond the acute phase, with 3% dying in hospital and 42% after discharge, indicating most burn-related deaths occur long after injury. A 33-year cohort study found a long-term high risk of all-cause mortality after burns. Adjusted MRR for pediatric and young-to-middle-aged patients was 1.6 (95% CI:1.3~2.0) and 1.8 (95% CI:1.7~2.0), with 38% and 44% of deaths attributable to burns, indicating a 60% to 80% higher long-term death risk than the general population (19, 20). The systemic inflammatory response and immunosuppression from severe burns persist long-term, with patients showing a significantly increased cancer risk during follow-up (SIR 1.39) and remaining at higher risk for decades after injury (21). Randall et al. (22) found that burn patients had significantly higher inpatient service utilization and financial burden than the uninjured. Following adjustment, the hospitalization rate for the burn cohort was 2.48 times (95% CI: 2.33~2.65) that of controls, while costs were 2.77 times (95% CI: 2.58~2.98) higher, reflecting significant long-term health impacts. Inhalation injury, sepsis, multi-organ failure, systemic inflammatory response syndrome, systemic circulatory and metabolic disorders, malnutrition are the key risk factors leading to adverse prognosis. Compared with the general population, severe burn survivors exhibit a significantly higher proportion of medical consultations for psychological issues and remain at risk of serious mental health disorders throughout their lives (12), including post-traumatic stress disorder (PTSD), anxiety disorders, depression, and suicidal ideation (23, 24). Sleep disorders and shame persist for 5 years or even longer after burn patients are injured (25). Psychosocial factors significantly impact severe burn outcomes. Nearly half (43.4%) of severe burns occur during household/recreational activities, among these, 38.8% have a mental illness history, and 24.4% were under controlled substances at injury. These findings are particularly pronounced among patients with severe burns in Switzerland (26, 27). The process from acute injury to chronic disease begins with severe systemic disorders caused by burns themselves. Local tissue injury can rapidly spread into systemic inflammation, and may also develop into burn shock, triggering multi-system stress reactions involving the nervous system (28). Therefore, it is imperative to thoroughly investigate the mechanisms underlying major organ damage caused by severe burns and to formulate targeted intervention strategies. See **Figure 1** for details.

**Figure 1:**
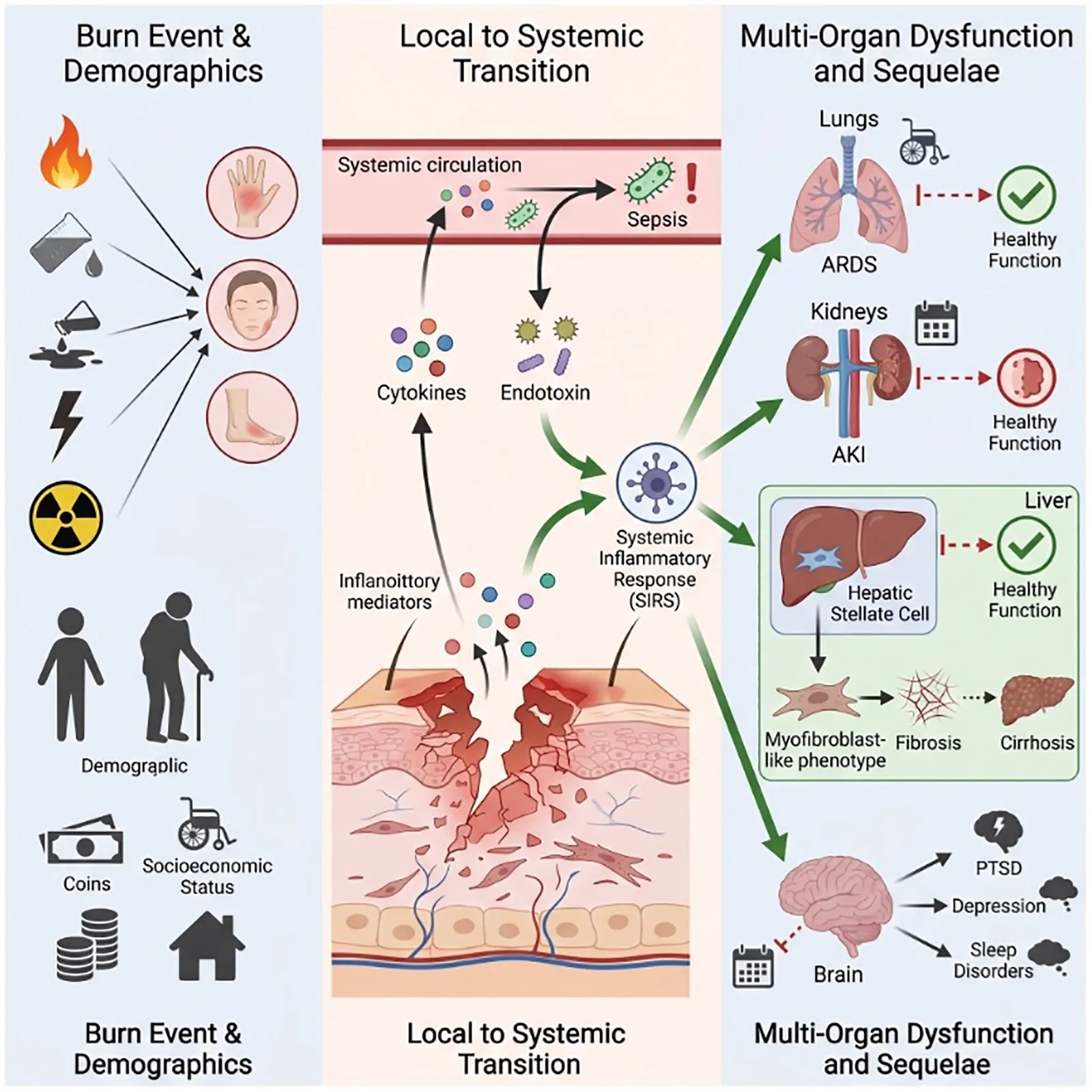
Global epidemiology of burns. Burn injuries stem from thermal, chemical, electrical and radiation sources. Elevated exposure risks arise from vulnerable populations (young children, the elderly), household/occupational environments, socioeconomic/mental disparities, and seasonal/temporal incidence peaks. Severe burns commonly damage the face, extremities and hands, accompanied by extensive TBSA third-degree wounds, inhalation injury, malnutrition and psychosocial risks. Acute complications include systemic inflammation, shock, sepsis, multi-organ dysfunction and early death. Surviving patients develop chronic hypermetabolism, persistent organ damage, nutritional, psychiatric and sleep disorders, resulting in high disability, mortality and permanent health sequelae. At the population level, severe burns contribute to substantial global burdens measured by incidence, mortality, YLDs, unequal geographic spread and large socioeconomic losses.

## 2 Pathophysiological mechanisms of burns

### 2.1 Inflammatory mechanisms

Post-burn inflammatory disturbance is the primary driver of organ damage (29). While burns are predominantly caused by thermal injury, they can also result from radiation, electrical shock, or chemical exposure, frequently accompanied by inhalation injuries and internal organ damage (30). Modern burn critical care establishes a core dichotomy of post-burn organ dysfunction including early sterile inflammation mediated injury driven by damage-associated molecular patterns (DAMPs) and late infection related organ failure dominated by pathogen-associated molecular patterns (PAMPs) (31). This classification provides a framework for both research and clinical decision-making.

Specifically, the first 72 hours after injury are a sterile period, driven by DAMPs released from thermal damage, burn shock, ischemia-reperfusion and necrotic tissue (32). Although the skin barrier is disrupted, organ dysfunction during this period is largely non-infectious. From day 3 to 5 onward, a septic phase develops, in which PAMPs from wound or pulmonary infection become dominant, often in an immunosuppressed host (33). This distinction is critical for both pathophysiology and clinical management.

Initial aseptic injury triggers an innate immune response (34). A transcriptomic study of 254 critically injured patients (167 with blunt trauma and 87 with burns) found that both pro-inflammatory mediators and anti-inflammatory pathways were upregulated over a 28-day period (29, 35). These findings indicate early-stage self-regulation, yet unchecked activation can trigger cytokine storm as well as systemic inflammatory response syndrome (SIRS) (36). See Figure Legend 1 for details.

The shift from sterile to infection-driven failure depends on necrotic tissue load, immune exhaustion, and iatrogenic factors. In porcine models, persistent complement and inflammatory cell infiltration in burn wounds and the heart indicate systemic involvement (37). However, if the initial DAMP response persists due to full-thickness burns, delayed eschar removal, or large TBSA, the necrotic environment sustains DAMP release and promotes bacterial growth. Concurrently, innate immunity becomes constrained. Chemokine recruitment increases (36), but neutrophil chemotaxis, phagocytosis, and oxidative burst decline, while lymphocyte apoptosis increases, leading to immunosuppression that favors PAMP-driven sepsis. Burns are not only acute injuries but also chronic conditions, with immune dysfunction continuing after wound closure (38), which lowers the threshold for septic transition.

Macrophage polarization illustrates the need for balanced immune regulation. Early M1 macrophages perform essential defense functions, including phagocytosis of necrotic debris and control of local infection (39) Without this response, uncontrolled infection can be fatal (40). However, a timely switch to the M2 pro-repair phenotype is equally critical (4, 41) M2 macrophages secrete growth factors (PDGF, VEGF, IGF-1) and pro-resolving mediators (resolvins, lipoxins) clear apoptotic cells, and promote angiogenesis and epithelial proliferation, thus supporting tissue repair (42). If this M1-to-M2 transition is delayed or incomplete, the host may remain in a hyperinflammatory or immunoparalyzed state, leading to multi-organ dysfunction syndrome (MODS) (43). Pathway analyses of leukocyte transcriptomes have identified integrin signaling, leukocyte extravasation, Th17-eIF2 stress recovery, and transcription factors (FOXO6, TEAD3, SUPT3H) as regulators of this balance (44). Thus, the balance among early pro-inflammatory responses, subsequent anti-inflammatory reactions and tissue regeneration requires strict regulation, whereas disruption at any time-point may hinder wound healing and raise infection risk (45). Therefore, the transcriptional, epigenetic, and metabolic programs controlling immune cell function warrant further study, especially in relation to the sterile-to-septic transition.

### 2.2 Tissue ischemia-reperfusion injury

Ischemia-reperfusion injury (IRI) is a pathological process in which blood flow restoration after ischemia exacerbates cellular damage due to oxygen free radicals, calcium influx, and inflammatory cell activation, observed in conditions like myocardial infarction, stroke, and organ transplantation (46). Severe burns commonly induce IRI through mechanisms such as energy metabolism disorders, oxidative stress, calcium overload, and inflammatory responses. IRI affects local tissues and indirectly impacts organs like the heart, lungs, and kidneys. For example, DAMPs from intestinal IRI after burns trigger systemic inflammation, leading to acute respiratory distress syndrome (ARDS) and acute kidney injury (AKI) (47), which are linked to microcirculatory disturbances, increased vascular permeability, hemoconcentration, and inadequate tissue perfusion from the burn (48). The severity of damage correlates with burn area and depth, potentially causing MODS (49). This is mainly due to early fluid resuscitation’s dual nature, which recovers blood volume and improves microcirculation but may worsen tissue injury with abrupt reoxygenation (50). This process induces massive oxygen free radical release and initiates inflammatory cascades. The ischemic period involves impaired mitochondrial function and disrupted energy metabolism. After reperfusion, activation of the xanthine oxidase system and mitochondrial electron leakage increase ROS production, triggering calcium overload, inflammatory signaling, and cell death (51). The ischemic phase causes tissue hypoxia, reduced ATP, and calcium accumulation, setting the stage for reperfusion injury. Reperfusion then stimulates ROS production, attacking cell membranes, proteins, and DNA (52, 53). Calcium influx activates proteases, disrupting the cytoskeleton (54). Neutrophils release ROS and enzymes damaging the endothelium (55). ROS activates immune cells (56), among them, M1 macrophages have pro-inflammatory effects, while M2 macrophages have anti-inflammatory and tissue-repairing effects (57). Oxidative stress and early sterile inflammatory responses jointly lead to mitochondrial dysfunction, which in turn triggers the activation of various programmed cell death pathways, ultimately resulting in an imbalance between tissue damage and repair (58, 59). Mitochondrial dysfunction and ROS activate necrosis pathways, with apoptosis and necrosis worsening tissue damage (60, 61). PANoptosis, involving the PANoptosome complex, simultaneously activates pyroptosis, apoptosis, and necroptosis (62). Acute liver injury from intestinal IRI after burns correlates with suppression of the Nrf2-ARE pathway, which normally mitigates oxidative damage but is impaired post-burn (63). Ischemia-reperfusion from severe burns drives MODS, and while fluid resuscitation restores perfusion, it can cause secondary injury via ROS and inflammatory cytokines. Kidneys show increased oxidative stress and apoptosis, and systemic inflammation and oxidative stress may lead to MODS, a major cause of death in burn patients (64).

### 2.3 Coagulation disorders

Coagulation system is a key defense mechanism for body stability. Severe burns often cause complex functional disorders, not just hypercoagulability or hypocoagulability, but a dynamic, staged pathological process involving multiple systems (65). The occurrence of such disorders is linked to serious initial injury and systemic pathological reactions. Extensive burns damage tissues and vascular endothelial cells, releasing tissue factors that activate exogenous coagulation pathways, initiating the coagulation cascade (66). Hypovolemic shock after burns causes tissue hypoperfusion, and hypoxia, metabolic acidosis, and hypothermia form the “triad of death,” leading to coagulation disorders and increased mortality risk (67).

Burns, as strong stressors, trigger systemic inflammation and release inflammatory mediators like TNF-*α* and IL-6, which are central to coagulation dysfunction post-burn (68). These cytokines upregulate tissue factor (TF) on endothelial cells and monocytes while downregulating thrombomodulin (TM), promoting thrombin production and weakening anticoagulation (69). Inflammatory environments stimulate plasminogen activator inhibitor-1 (PAI-1) release, inhibiting fibrinolysis and impairing microthrombus removal (70). Meanwhile, inflammation and fibrinolytic inhibition exacerbate coagulation dysfunction. Fluid resuscitation, though necessary, dilutes clotting factors and platelets, exacerbating this dysfunction. Severe burn patients often face sepsis and inhalation injury, both negatively impacting prognosis (71). Inhalation injury can cause local lung coagulation issues and sepsis may lead to disseminated intravascular coagulation (DIC), harming coagulation in severe burn patients. These disorders show stage-dependent changes, starting with a hypercoagulable state marked by increased thrombin and fibrinogen (72), detectable via thromboelastogram (TEG) parameters like *α* angle and maximum amplitude (MA) (73). Simultaneously, the body’s anticoagulant system is rapidly consumed, with key substances like antithrombin III (AT III), protein C (PC), and Protein S (PS) decreasing early, causing loss of negative feedback in over-activated coagulation (74). Complex variations take place within the fibrinolytic system afterwards, with sharp PAI-1 elevation inhibiting fibrinolysis in the post-burn hypercoagulable stage. The suppressed tissue plasminogen activator (t-PA) and urokinase-type plasminogen activator (u-PA) activity ultimately results in functional disturbance (75). Secondary hyperfibrinolysis often occurs after this stage, with damaged endothelial cells releasing large amounts of t-PA. Meanwhile, declining PAI-1 levels relieve inhibition of the fibrinolytic system. In patients on extracorporeal membrane oxygenation (ECMO), elevated plasma t-PA precedes bleeding, indicating excessive t-PA release is key to fibrinolysis hyperfunction and hemorrhage risk (76). Hyperfibrinolysis degrades fibrin or fibrinogen, producing fibrin degradation products (FDPs) with anticoagulant properties that inhibit fibrin polymerization and impair platelet function, leading to serious bleeding tendency (77). Platelet count and function change significantly, often decreasing rapidly in early burns. This is due to collagen exposure activating platelets, causing local adhesion and microthrombosis for wound healing (78), and systemic inflammation promoting platelet interaction with white blood cells and endothelial cells, further reducing circulating platelets (79). In a sustained activation state, platelets often show reduced reactivity to agonists like adenosine diphosphate (ADP), collagen, and thrombin, along with impaired aggregation, likely due to granule depletion, signal desensitization, and receptor downregulation (80). This dysfunction may raise bleeding risk in severe burns or sepsis (81). Burns disrupt endothelial cell homeostasis, leading to severe injury that triggers coagulation issues and cytokine storms, with endothelial barrier loss and phenotype changes forming a complex network that increases the risk of microcirculatory disturbances and organ failure (82).

The uncontrolled pathological process leads to serious clinical outcomes, with DIC as the most severe consequence (83). Microvascular thrombosis causes microcirculation failure and organ ischemia, contributing to MODS (66). Coagulation dysfunction severity correlates with organ failure, and prolonged activated partial thromboplastin time (APTT), increased international standardized ratio (INR), and hypofibrinogenemia are linked to high mortality. Coagulation dysfunction is a strong predictor of death (84), with INR increase, APTT extension, thrombocytopenia, D-dimer rise, and soluble TREM-like transcript-1 (sTLT-1) decrease as short-term risk factors, indicating an irreversible final stage (85, 86).

### 2.4 Neuroendocrine disorders

Severe burns trigger systemic responses beyond local damage, with neuroendocrine disturbances key to prognosis (40). Research focuses on hypothalamus-pituitary-adrenal (HPA) axis dysfunction, sympathetic-adrenal medulla (SAS) overactivation, thyroid abnormalities, and insulin resistance (87). The neuroendocrine system, a stress regulator, changes significantly post-burn, causing compensatory reactions despite potential secondary injuries (88). Severe burns rapidly activate the HPA axis, triggering acute neuroendocrine responses (89). Hypothalamic corticotropin-releasing hormone (CRH) neurons increase secretion, stimulating adrenocortical hormone (ACTH) release from the anterior pituitary immediately after stressors emerge, even before systemic reactions like fever. ACTH then stimulates adrenal cortex to release glucocorticoids (GCs), such as cortisol in humans and corticosterone in rodents, with GC levels rising quickly (90). Acute HPA axis overactivation is a protective response to severe trauma, mobilizing energy and inhibiting inflammation. For example, in acute coronary syndrome, it is a normal physiological reaction (91). This reaction carries dual effects, with increased GC contributing to SIRS regulation and tissue-damage prevention following burn injury, but sustained high GC levels cause metabolic side effects and may increase infection risk (92). HPA axis activation drives *α*-MSH secretion in inflammatory bowel disease, aiding acute pathogen clearance but risking chronic inflammation, reflecting stress timing effects (91). After severe burns, acute HPA activation requires careful control to balance benefits and risks. In chronic burns, HPA overactivation shifts to functional inhibition, causing Relative adrenal insufficiency (RAI) with low cortisol or reduced stress reactivity (93). Studies indicate that reduced HPA axis function, observed in coronary heart disease and hypertension, predicts higher cardiovascular risk (91). In chronic severe burns, persistent inflammation and stress lower HPA axis reactivity via negative feedback, involving altered glucocorticoid receptor (GR) expression and function, such as abnormal GR phosphorylation in brain regions like the hypothalamic paraventricular nucleus. Chronic stress increases phosphorylation at Ser203 (reducing activity) and decreases it at Ser211 and Ser234, impairing GR transcriptional activity and feedback (94). Upregulation of FKBP5 disrupts GR translocation and DNA binding, thereby preventing high GC levels from inhibiting CRH and ACTH secretion and establishing a vicious cycle with inflammation. After chronic stress, pituitary ACTH cell excitability remains inhibited for weeks due to transcription reprogramming, indicating persistent HPA axis dysfunction (95). The mild cortisol decrease, weakened circadian rhythm, and slow HPA response in chronic fatigue syndrome reflect this dysfunction (96). In severe burn patients, HPA axis dysfunction includes adrenal insufficiency and glucocorticoid resistance, linked to impaired negative feedback. The interaction between sustained inflammation and HPA axis dysfunction is a key pathophysiological factor affecting long-term prognosis (97).

Excessive activation of the sympathetic-adrenal medulla (SAM) system after severe burns sharply raises catecholamine levels, linked to multi-organ injuries. For instance, in pheochromocytoma patients, excessive catecholamine levels can cause Takotsubo cardiomyopathy and cardiogenic shock (98, 99). After burns, plasma norepinephrine and adrenaline peak in the acute phase (one week post-burn) (100). Excessive catecholamine induces hemodynamic overload via *β*-adrenergic receptors, leading to tachycardia, hypertension, and increased myocardial oxygen consumption (101). Animal studies show that continuous infusion of exogenous catecholamine (e.g., norepinephrine) causes severe tachycardia and ventricular dysfunction, with norepinephrine being more toxic than epinephrine (101). Over-activation of the sympathetic nervous system (SNS) leads to acute cardiovascular injury and down-regulation of *β*-adrenergic receptors, weakening myocardial response to catecholamines. This desensitization is seen in pheochromocytoma and burn patients, reducing reactivity to *β*-agonists (102, 103). Chronic sympathetic hyperexcitation is associated with myocardial injury, arrhythmias, and post-burn sympathetic remodeling that may cause neuropathic pain (104). Mechanisms involve peripheral and central sensitization, abnormal *α*1-adrenergic receptor expression in dorsal root ganglia, and pro-inflammatory cytokine release e.g., IL-6, TNF-*α* worsening pain sensitivity (105). These findings help understand long-term effects of Neuroendocrine dysfunction after Burns. HPT axis inhibition is key to burn progression. Pro-inflammatory cytokines like IL-6, TNF-*α* reduce TRH and TSH release, lowering thyroid hormone synthesis (106). This inhibition decreases metabolic rate and energy use, lasting into recovery. Sustained low serum T3 is an independent risk factor for burn mortality (107). In severe burns, low T3 syndrome patients have over 3 times higher 28-day mortality than those with normal thyroid function, and T3 levels inversely correlate with APACHE II score (108). This may result from T3 deficiency disrupting energy metabolism in vital organs, potentially causing organ failure (109). Low T3 syndrome reflects thyroid dysfunction and an adaptive stress response, but prolonged persistence can lead to multi-organ dysfunction (110).

### 2.5 Metabolic disorders

Severe burns often cause prolonged hypercatabolism, with hypermetabolism as a key feature, marked by increased oxygen consumption, energy use, and insulin resistance (111). If uncontrolled, metabolic demands rise, leading to catabolism of muscle, bone, and fat, and adverse outcomes like organ dysfunction or death (112). After burns, the HPA axis and sympathetic-adrenal medulla system are strongly activated, raising stress hormones like cortisol and catecholamines (113). After severe burns, high metabolism and hormone actions enhance gluconeogenesis and glycogen breakdown, while peripheral glucose uptake is inhibited, causing hyperglycemia (114). Insulin signaling is severely impaired, with resistance in skeletal muscle, liver, and adipose tissue (115). Pathways like IRS-PI3K-Akt are disrupted, reducing glucose transporter 4 (GLUT4) transport and glucose uptake (116). Post-burn suppression of insulin signaling leads to resistance in skeletal muscle, liver, and adipose tissue. Mechanistically, this entails impaired signaling through the IRS-PI3K-Akt pathway, thereby reducing GLUT4 translocation and glucose uptake (117). Hyperactive neuroendocrine function accelerates glycogenolysis, inhibits insulin secretion, and induces peripheral insulin resistance, causing severe metabolic disorders in burn patients (118). This metabolic abnormality raises the risk of multi-organ dysfunction, notably myocardial energy exhaustion and depleted liver glycogen reserves (119). High metabolism also features browning of subcutaneous white adipose tissue (WAT), a key regulatory factor (120). Adipose tissue, once seen as a storage depot, is now recognized as an endocrine organ mediating both metabolic diseases and systemic dysfunction after trauma like severe burns (121). Thermal injury induces chronic *β*-adrenergic response with lipolysis, macrophage infiltration, and IL-6-mediated WAT browning (122). The effects of these physiological changes on adipose tissue have only recently been elucidated (123). Such metabolic disturbances often cause cardiac dysfunction and multi-organ failure via lipolysis and proteolysis.

Severe burns also trigger a chronic hypermetabolic state lasting years post-discharge, implying other internal mechanisms (111). Severe burns induce systemic oxidative stress linked to IRI from fluid resuscitation, neuroendocrine stress, and fluid imbalance (124). This stress worsens tissue damage and may impair neuroendocrine function by affecting the hypothalamus-pituitary-thyroid (HPT) and hypothalamic-pituitary-gonadal (HPG) axes (123). Wound healing is prolonged in severe burns, with a marked and sustained increase in basal energy expenditure, intensifying stress responses and catabolic effects (125). At the tissue level, mitochondrial dysfunction drives post-burn hypermetabolism, mediated by mechanisms in skeletal muscle and adipose tissue that favor heat generation over ATP synthesis (126, 127). Energy supply and oxidative stress are linked through mitochondrial regulation, and conventional biomaterials fail to address both ATP demand and mitochondrial dysfunction in severe burns (128). Prolonged hypermetabolism poses long-term health threats. Burn survivors have a higher incidence of endocrine disorders and musculoskeletal injuries within three years of their injury (129, 130). Other long-term effects include cardiac dysfunction, increased infection risk (131), mental health disorders (132), and elevated mortality (133). Further research is lacking on the biological mechanisms behind long-term burn-induced systemic disturbances and their role in complications.

Clinically, animal models, due to *in vitro* limitations, test post-burn interventions and therapies to modulate the hypermetabolic state. These include nutritional support, early debridement, and growth hormone (134).

This trigger induces catabolic responses in multi-organs, including muscle proteolysis, adipose lipolysis, liver glycogenolysis, and fatty infiltration (135), increased intestinal permeability, and higher renal, cardiac, and metabolic stress. These responses cause insulin resistance, hyperglycemia, hyperlipidemia, and mitochondrial dysfunction, leading to severe energy deficiency (136) and, with excessive inflammation, a higher risk of sepsis and organ failure-a key mechanism in burn mortality (137).

### 2.6 Ferroptosis

In this section, we aim to synthesize the current research landscape of ferroptosis and its potential value in organ injury caused by severe burns. Ferroptosis is a newly discovered form of regulatory cell death (RCD). Its core feature is the lethal accumulation of lipid peroxides that are dependent on iron ions in the cell membrane system, this mode of death is essentially different from other forms of cell death such as apoptosis and necrosis (138). The regulation process of ferroptosis mainly involves many biological pathways, including iron metabolism, redox homeostasis and lipid metabolism (138). Through a systematic review, Noguchi and colleagues found that iron metabolism disorders are closely associated with the formation and activation of lipid peroxides (139). This process increases cell membrane permeability, thereby promoting lipid peroxidation propagation and ferroptotic cell death (140). Recent studies have revealed that ferroptosis plays a critical role in organ damage caused by various triggers, including heart failure, alkali burns to the cornea, IRI, neurodegenerative diseases, and chemotherapy-related damage (141). Overall, the currently available research evidence primarily comes from preclinical animal models and some relevant human data. These findings aim to explore the potential role of ferroptosis in tissue damage. However, its efficacy in burn patients has not yet been fully validated. Therefore, further research into the role of ferroptosis in burn-induced organ damage holds significant scientific value.

Following severe burns, iron metabolism disorders primarily occur during the processes of iron uptake, storage, release, and transport. A retrospective cohort study analyzing iron metabolism data in adult burn patients found that these patients exhibited varying degrees of impaired iron utilization, primarily manifested as transferrin saturation <20% and abnormal intracellular accumulation of iron ions, which in turn led to ferroptosis (141, 142). The underlying mechanism involves the upregulation of transferrin receptor (T*f*R) expression, which promotes cellular uptake of iron ions, while the downregulation of ferritin heavy chain (FTH1) and light chain (FTL) expression reduces iron storage capacity. This ultimately leads to elevated intracellular concentrations of free Fe^2+^, which continuously triggers the Haber-Weiss reaction and the Fenton reaction (140). Free iron ions can induce the massive production of ROS via the Fenton reaction, further exacerbating lipid peroxidation and ultimately driving the ferroptosis process.

Lipids are an essential component of the cell membrane system and are involved in numerous cellular physiological functions, such as energy supply and signal transduction. Differences in lipid composition form the basis for the diversity of organelles and various subcellular structures (143). Experiments using a rat skin burn model have shown that phospholipids (PLs) are one of the key lipid classes that determine sensitivity to ferroptosis within cells, with the metabolism of polyunsaturated fatty acid phospholipids (PUFA-PLs) being regulated by acyl-CoA synthase long-chain family member 4 (ACSL4) and lytic phosphatidylcholine acyltransferase 3 (LPCAT3) (144). In the classical molecular pathway of ferroptosis, phospholipids on the cell membrane that are rich in polyunsaturated fatty acids (PUFAs)—such as PUFA-PLs generated by ACSL4—are prone to peroxidation under the action of free radicals (139). Fenton reaction-driven peroxidation of PUFA-PLs (primarily arachidonic acid, AA) can lead to excessive accumulation of membrane phospholipid peroxides (PLOOHs). The abnormal accumulation of lipid peroxidation end products, such as malondialdehyde (MDA) and 4-hydroxynonenal (4-HNE), is considered an important concomitant phenomenon of ferroptosis at the cellular and tissue levels (145). These products compromise cell membrane integrity and ultimately lead to cell lysis (146).

Intracellular antioxidant systems can directly neutralize lipid peroxidation reactions and serve as important defense mechanisms against ferroptosis. Glutathione peroxidase 4 (GPX4) serves as the pivotal enzyme suppressing ferroptosis, and it sustains intracellular redox homeostasis through the reduction of lipid peroxides (147). In animal models following severe burns, both the expression levels and enzymatic activity of GPX4 show a significant downregulation, which prevents the effective clearance of lipid peroxides. This, in turn, promotes the rapid accumulation of PLOOHs on the cell membrane and ultimately induces ferroptosis (148). Previous studies based on preclinical models have shown that GPX4 protein expression is downregulated in skin tissue following burns, a phenomenon consistent with the trends observed in changes of ferroptosis-related biochemical markers (such as MDA and 4-HNE) in animal tissues (149). However, caution is warranted when directly extrapolating the aforementioned molecular pathways to human patients with severe burns—markers such as MDA and 4-HNE are established indicators of systemic oxidative stress in clinical samples, and direct *in vivo* evidence in humans remains limited to definitively confirm the occurrence of this cascade in burn patients.

### 2.7 Endoplasmic reticulum stress

Previous studies in rodents have shown that the accumulation of misfolded proteins in the endoplasmic reticulum triggers a stress response. Severe burns disrupt the homeostasis of the endoplasmic reticulum, thereby activating the unfolded protein response (UPR). Persistent stress induces pro-apoptotic signaling, leading to tissue damage and cell death, which in turn triggers MODS (150). The mechanisms of ERS induction in severe burns are complex, involving four key factors. First, reduced blood flow causes insufficient perfusion, triggering ischemia-hypoxia and metabolic disturbances that shift energy production toward anaerobic glycolysis, thereby reducing ATP synthesis (151).

Experiments in mice have shown that HIF-1*α* can indirectly regulate the stress response through UPR-related genes (152). Second, Oxidative stress and ROS accumulation occur during ischemia-reperfusion in burn wounds (153). ROS damage ER lipids and proteins, disrupting redox homeostasis, and activate UPR receptors like PERK and inositol-requiring enzyme 1 alpha (IRE1*α*), amplifying ER stress (154). Mouse models have demonstrated that the aforementioned process interacts with mitochondrial dysfunction, creating a vicious cycle. Experimental studies in rats have confirmed that reactive oxygen species (ROS) can also enhance the proapoptotic effects of endoplasmic reticulum stress (ERS) by activating the c-Jun N-terminal kinase (JNK) pathway (155). Third, calcium homeostasis imbalance occurs. Studies of burn patients have shown that burns damage cell membranes, leading to SERCA dysfunction and excessive activation of IP3R channels, which in turn causes leakage or depletion of the endoplasmic reticulum calcium pool (156). This change impairs calcium-dependent chaperones, triggers cytoplasmic calcium overload, and activates proteases, disrupting UPR signaling (157, 158). Fourth, DAMPs released by damaged tissues trigger sterile inflammation, leading to the production of pro-inflammatory cytokines. These cytokines interact with the UPR, thereby exacerbating the protein overload in the endoplasmic reticulum. The ERS also induces a systemic inflammatory cascade by activating the NLRP3 inflammasome, ultimately affecting multi-organs (159).

ERS progression is mainly regulated by UPR’s three core pathways. PERK activates after glucose-regulated protein 78/immunoglobulin heavy chain binding protein (GRP78/BiP) dissociates, inhibiting protein translation via eIF2*α* phosphorylation while selectively promoting activation of transcription factor 4 (ATF4) mRNA translation (160).

This pathway moderately increases the expression of antioxidant genes. However, sustained stress induces ATF4 to express C/EBP homologous protein (CHOP), thereby triggering apoptosis. Once activated, the IRE1*α* pathway exerts dual enzymatic functions as both a ribonuclease and a kinase. This property is documented within the findings of Ilaria et al. (161) Are consistent with this conclusion. On the one hand, it produces XBP1s by cleaving XBP1 mRNA, thereby upregulating the expression of endoplasmic reticulum-associated degradation (ERAD) genes to promote adaptive responses (162). On the other hand, it recruits TRAF2 to activate the ASK1-JNK/p38 signaling pathway and works in concert with CHOP to promote apoptosis (163). Activating transcription factor 6 (ATF6), which is cleaved in the Golgi apparatus, acts synergistically with XBP1s to enhance the expression of molecular chaperones and ERAD genes, thereby exerting a cytoprotective effect (164). Under pathological conditions like extensive burns, an imbalance among the three signaling pathways due to ATF6 overactivation or antagonism by PERK/IRE1*α* pro-apoptotic signaling may be a key mechanism for multi-organ dysfunction (165).

Sustained ERS activation triggers apoptosis through CHOP, IRE1*α*-JNK, and caspase-12/4/5 pathways, while also regulating autophagy, which can clear abnormal proteins and reduce ERS. Excessive or dysregulated autophagy worsens damage and interacts with inflammation, causing a harmful cascade (165). Experiments in mice have shown that ERS triggers mitochondrial calcium overload and loss of membrane potential via mitochondrial-endoplasmic reticulum contact sites (MAMs), thereby leading to a vicious cycle between endoplasmic reticulum stress and mitochondrial dysfunction (166). *In vitro* cell models show that ERS disrupts endoplasmic reticulum–Golgi transport, affecting lysosomal stability and autophagy flux, thereby exacerbating stress-induced damage (167). Experimental studies have shown that ERS in pancreatic *β*-cells following severe burns leads to abnormal insulin secretion, but the inhibitor 4-phenylbutyric acid (4-PBA) can improve this condition (168). In a corneal alkali burn model, endoplasmic reticulum stress was involved in retinal ganglion cell apoptosis, and its inhibition reduced inflammation and protected retinal structure (169). Following burns, endoplasmic reticulum stress in adipose tissue is linked to metabolic dysfunction, and propranolol can improve function by alleviating this stress (111). These findings indicate endoplasmic reticulum stress is key in post-burn multi-organ dysfunction.

## 3 Burn-induced damage to vital organs

The pathophysiological effects of burns extend far beyond the destruction of the local skin barrier. It represents a profound systemic stressor that can affect nearly every organ system. Yet its global health burden remains critically underappreciated. See **Figure 2**, **Table 1** for details.

**Figure 2:**
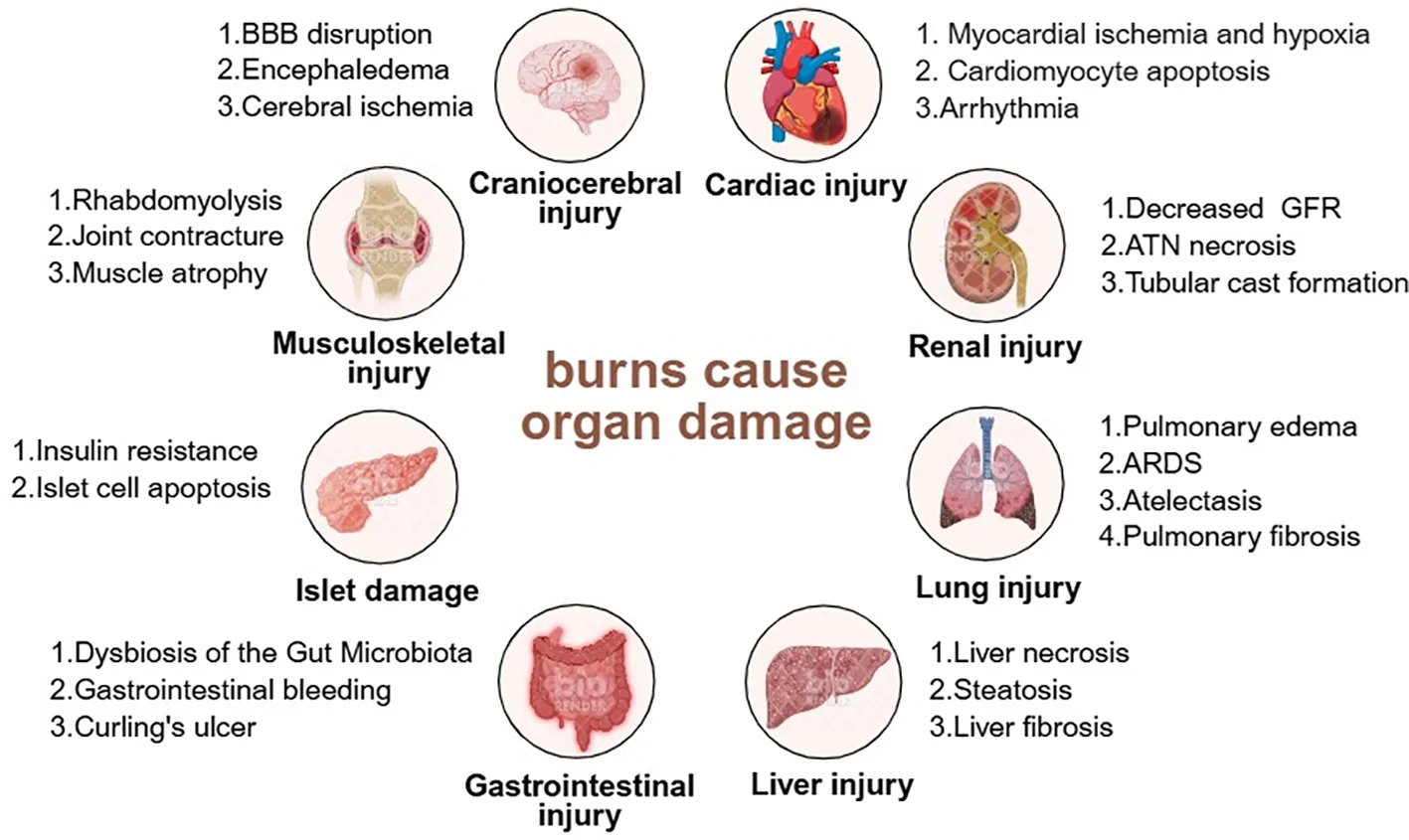
Schematic diagram of burn-induced damage to vital organs. Severe burns trigger synergistic systemic pathologies including unregulated inflammation, hypermetabolism, neuroendocrine activation, endothelial injury and tissue hypoperfusion, leading to secondary damage to non-heated organs. This figure depicts lesions of eight key organ systems post major burns, including brain, heart, kidney, lung, liver, GI tract, pancreatic islets and musculoskeletal system.

**Table 1:** Overview of the temporal course of organ injury following burns.

Organs affected	Acute phase		Subacute period		Chronic phase	
	Dominant mechanism	Interventions	Dominant mechanism	Interventions	Dominant mechanism	Interventions
Heart	Catecholamines induce calcium overload, open mPTP and collapse membrane potential, causing myocardial apoptosis/necrosis. TNF-*α* and IL-1*β* reduce myofilament calcium sensitivity and depress myocardial function, while sustained *β*-adrenergic receptor activation disrupts signaling pathways.	Serum Cardiac Enzymes (CK-MB, cTnI). Early detection of myocardial injury. Monitoring of LVEF, cardiac output, and volume status. Prevention of acute heart failure.	Persistent immune dysregulation (a shift from a pro-inflammatory to an immunosuppressive state). Mitochondrial dysfunction energy metabolic imbalance calcium homeostasis dysregulation.	Early debridement, immune modulation, mitochondria-targeted drug therapy, antioxidant therapy, reducing metabolic burden, energy and nutritional support therapy, calcium homeostasis regulation.	Sustained sympathetic activation, chronic oxidative stress, TGF-*β*/Smad pathway-induced myocardial fibrosis, persistent mitochondrial dysfunction and energy metabolism disorders.	Cardiac function, exercise intolerance, and chronic arrhythmias require long-term monitoring. Management of myocardial fibrosis and long-term adverse cardiovascular events. Control risk factors like hypertension and hyperlipidemia.
Liver	DAMPs trigger sterile inflammation, early hypoperfusion, hepatic sinusoidal stasis, kupffer cells (KCs) activation, oxidative stress multiple forms of programmed cell death (apoptosis, necroptosis, pyroptosis, ferroptosis).	Fluid resuscitation, continuous monitoring of transaminases (AST/ALT), optimizing fluid resuscitation, wound care.	Programmed cell death, post-resuscitation IRI, persistent inflammatory response, delayed resuscitation, Persistent activation of KCs, hypermetabolic state, dyslipidemia, insulin resistance.	Ferroptosis antagonist therapy. Continuous monitoring of liver blood flow and function. Development of targeted immunomodulatory approaches. Control of infection sources and reduction of the risk of secondary infections	Activation of hepatic stellate cells (HSCs) fibrosis initiation, impaired sinusoidal microcirculation, PAMP-mediated inflammation and metabolic disorders (glucose, lipids, hypoalbuminemia, impaired detoxification).	Long-term dynamic monitoring of liver function markers. Assessment of nutritional and metabolic status. Management of cholestasis and impaired bile synthesis.
Kidney	Sensitivity to ischemia and hypoxia, hypovolemia, renal vasoconstriction (sympathetic/RAS activation), rhabdomyolysis, hemolysis, pigment toxicity, resuscitation-related factors, nephrotoxic drugs.	Early, goal-directed fluid resuscitation. Avoid the use of nephrotoxic medications. Monitor relevant laboratory parameters to identify and assess AKI early. Early CRRT (if necessary).	High metabolic rate, recurrent infections, immune dysregulation, IRI, accumulation of nephrotoxins, inflammation and immune imbalance TGF-*β*/Smad and other fibrogenic signaling pathways are activated, mitochondrial dysfunction and oxidative stress.	Initiate nutritional support as early as possible, maintain renal perfusion, initiate early antimicrobial therapy, reduce drug-induced AKI reduce ROS and protect mitochondrial function.	Antimicrobial−related nephrotoxicity, chronic inflammation and endothelial/microcirculatory dysfunction, progressive fibrosis, CKD, and secondary immune responses and MODS triggered by infection, sepsis, or septic shock.	Antimicrobial stewardship, control of systemic inflammation, support of microcirculatory function, and innovative research on extracorporeal blood purification and immunomodulatory interventions.
Lung	Direct thermal injury to the airway mucosa, inhalation injury, sterile inflammation, and mucosal congestion, edema, and necrosis.	Early aggressive airway management, supportive care, and antimicrobial therapy.	Inhalation injury-induced persistent pulmonary dysfunction, persistent neutrophil infiltration into lung tissue, and limited innate immune response.	Airway humidification and bronchodilators, physical mucus clearance, local or systemic anti-inflammatory therapy, and early debridement.	Impaired ciliary clearance, reduced phagocytosis by alveolar macrophages, local immunosuppression, bacterial colonization and proliferation, and infectious inflammatory response and sepsis.	Antimicrobial therapy, supportive care, prevention of complications such as sepsis and MODS.
Brain	DAMPs trigger sterile inflammation and SIRS, disruption of the BBB/BSCB, plasma leakage, and cerebral edema, impaired mitochondrial function and an acute ATP energy crisis, and sympathetic overactivation, cholinergic pathway inhibition.	Protecting the BBB, correcting metabolic disorders, precision fluid resuscitation, and targeted treatment strategies.	Persistent neuroinflammation with glial activation. Incomplete BBB repair and ongoing microvascular dysfunction. Early neurocognitive impairment and emotional dysregulation.	Anti-inflammatory and immunomodulatory therapy, optimized calcium management, antioxidant therapy, early rehabilitation training, and early cognitive and psychosocial screening.	Persistent energy and metabolic issues, neuronal and glial swelling, systemic stress, and cerebral edema. Organ dysfunction from infection and sepsis. Long-term neurocognitive decline, mood disorders, and chronic pain.	Long-term infection treatment, rehabilitation exercises, psychological support, pain management, neurological monitoring, multidisciplinary follow-up, healthy lifestyle promotion, and early cognitive intervention.
Gastrointestinal tract	Splanchnic vasoconstriction and ischemia-reperfusion injury, capillary leakage and microthrombosis, catecholamine-induced mucosal ischemia and cytokine storm, and tight junction disruption, REG3G downregulation, and mucin inhibition.	Correct hemodynamic disturbances, improve microcirculatory dysfunction, inhibit inflammatory cascades, and restore the pro-inflammatory/anti-inflammatory balance.	SIRS and neuroendocrine dysregulation (HPA axis, sympathetic overactivity), gut microbiota dysbiosis, intestinal barrier disruption and bacterial translocation, and M1 polarization of Kupffer cells.	Early fluid resuscitation, anti-inflammatory therapy, endotoxin adsorbers, pharmacological intervention, and early nutritional support.	Persistent inflammatory response, neuroendocrine dysfunction, gut microbiota dysbiosis, bacterial translocation, dysfunction of the gut-brain axis, and disruption of interactions among the gut, microbiota, and distant organs.	Drug therapy for ongoing digestive and absorptive issues, enteral nutrition with trace element supplementation, and routine nutritional monitoring and multidisciplinary follow-up.
Islet	Stress-induced hyperglycemia, systemic inflammation, which directly leads to *β*-cell dysfunction, oxidative stress and mitochondrial dysfunction.	Intensive insulin therapy, early continuous glucose monitoring, and correction of ischemia and hypoxia during shock phase.	Inflammatory cytokine storm (IL-1*β*, TNF-*α*) induces *β*-Cell apoptosis. Endoplasmic reticulum stress leads to *β*-cell dysfunction. Lipid browning and insulin resistance. Persistent stress-induced hyperglycemia. High metabolic state.	Anti-inflammatory treatment, precision insulin therapy, continuous dynamic monitoring of blood glucose levels	Progressive *β*-cell failure, mitochondrial dysfunction, chronic endoplasmic reticulum stress, impaired PI3K/Akt signaling, persistent hyperglycemia may cause microvascular complications.	Pharmacological intervention (insulin therapy), long-term blood glucose control and metabolic follow-up, and prevention and early detection of microvascular complications.
Musculoskeletal	Heat-induced protein denaturation, cell necrosis, microcirculatory ischemia and hypoxia, ATP depletion, lactic acid buildup, and rhabdomyolysis.	Early detection of muscle weakness, myalgia, and elevated CK levels; and monitoring for acute tubular necrosis.	Asymmetric muscle atrophy and prolonged immobilization or delayed rehabilitation exacerbate disuse atrophy of muscle groups and joint stiffness.	Posture management, development and implementation of personalized rehabilitation treatment, and use of orthoses for early intervention.	Persistent hypermetabolic and catabolic states, activation of the ubiquitin-proteasome pathway, NF-κB-mediated inflammation, mitochondrial dysfunction, oxidative stress, imbalance in RANKL/OPG and Wnt/β-catenin signaling, and TGF-β1/Smad2/3-mediated collagen deposition.	Long-term management of muscle atrophy and joint contractures, management of vitamin D deficiency and GH/IGF-1 axis dysfunction, and monitoring of ectopic ossification and fracture risk.

### 3.1 Brain injury and encephalopathy

Brain injury caused by severe burns is a serious complication of the central nervous system. This complication accounts for 16% of burn-related deaths, and significantly reduces the quality of life for survivors (170).

During the acute phase of burns, local tissue damage releases DAMPs, triggering sterile inflammation. In severe cases, this leads to SIRS (36). This disruption compromises the blood-brain barrier (BBB) and blood-spinal cord barrier (BSCB), which safeguard the central nervous system (CNS), thereby permitting the infiltration of inflammatory mediators (171). The loss of selective permeability of the BBB results in plasma leakage, subsequently inducing cerebral edema and cellular lesions (172). Metabolic disturbances triggered by SIRS can inhibit mitochondrial oxidative phosphorylation, resulting in decreased adenosine triphosphate (ATP) levels and lactic acid accumulation (173). Furthermore, SIRS can cause excessive sympathetic nervous system activation and inhibit cholinergic anti-inflammatory pathways, thereby further exacerbating cerebral inflammation (174). Clinically, this manifests as severe abnormalities in cerebral energy metabolism, cytotoxic cerebral edema, and impaired consciousness. If fluid resuscitation is improperly managed, it can more easily lead to increased intracranial pressure or even cerebral herniation, significantly increasing the risk of early mortality and adversely affecting short-term prognosis (175).

As the disease progresses, the body enters a flow phase that lasts from several months to several years, characterized by a persistent hypermetabolic response and chronic inflammation. This is the key driver of the transition from acute injury to chronic remodeling (112, 176). In addition, persistent lipid metabolism abnormalities often occur after burns, manifested as elevated triglycerides, decreased High-density lipoprotein (HDL), and an increase in small, dense Low-density lipoprotein (LDL). This atherogenic phenotype can persist for many years (112).

From a long-term perspective, studies have indicated that severe burns leading to systemic stress, hypoperfusion, or secondary infection can cause abnormalities in cerebral energy metabolism and insufficient ATP production, which are associated with cytotoxic cerebral edema (177). ATP deficiency impairs sodium-potassium pump function, leading to sodium retention and water influx, which in turn causes swelling of neurons and glial cells (178). The risk of hospitalization related to the nervous system is significantly elevated following burns (incidence ratio [IRR]=2.20, length of hospital stay is 3.25 times higher), and this risk in childhood can persist for up to 15 years after the injury, indicating that the systemic inflammation and immune dysfunction triggered by burns are not acute and self-limiting, but rather a long-term, persistent pathophysiological process (179). Furthermore, burn survivors often experience psychological and mental health issues, such as anxiety, depression, sleep disorders, cognitive decline, and PTSD, particularly among older adults. These issues can persist for months to decades after the acute phase, significantly reducing quality of life and increasing the risk of impaired social functioning (180). Some patients also experience long-term motor dysfunction, joint pain, and muscle weakness. These symptoms persist for an average of 17 years after injury (181). Moreover, secondary to initial nerve damage and altered neuronal firing patterns, hyperalgesia and neuropathic pain represent common long-term complications (182). Although initially thought to be related to psychological trauma, a clear pathophysiological link between burn-induced systemic stress and neurological dysfunction has now been established. Traumatic brain injury (TBI) is a serious complication following severe burns, while existing studies demonstrate that burn patients with concomitant TBI possess a markedly elevated mortality rate *OR* = 4.22 and unfavorable prognosis (183). Therefore, there is an urgent need to explore ways to improve the long-term prognosis of brain injury and the central nervous system following burns. See **Figure 3** for details.

**Figure 3:**
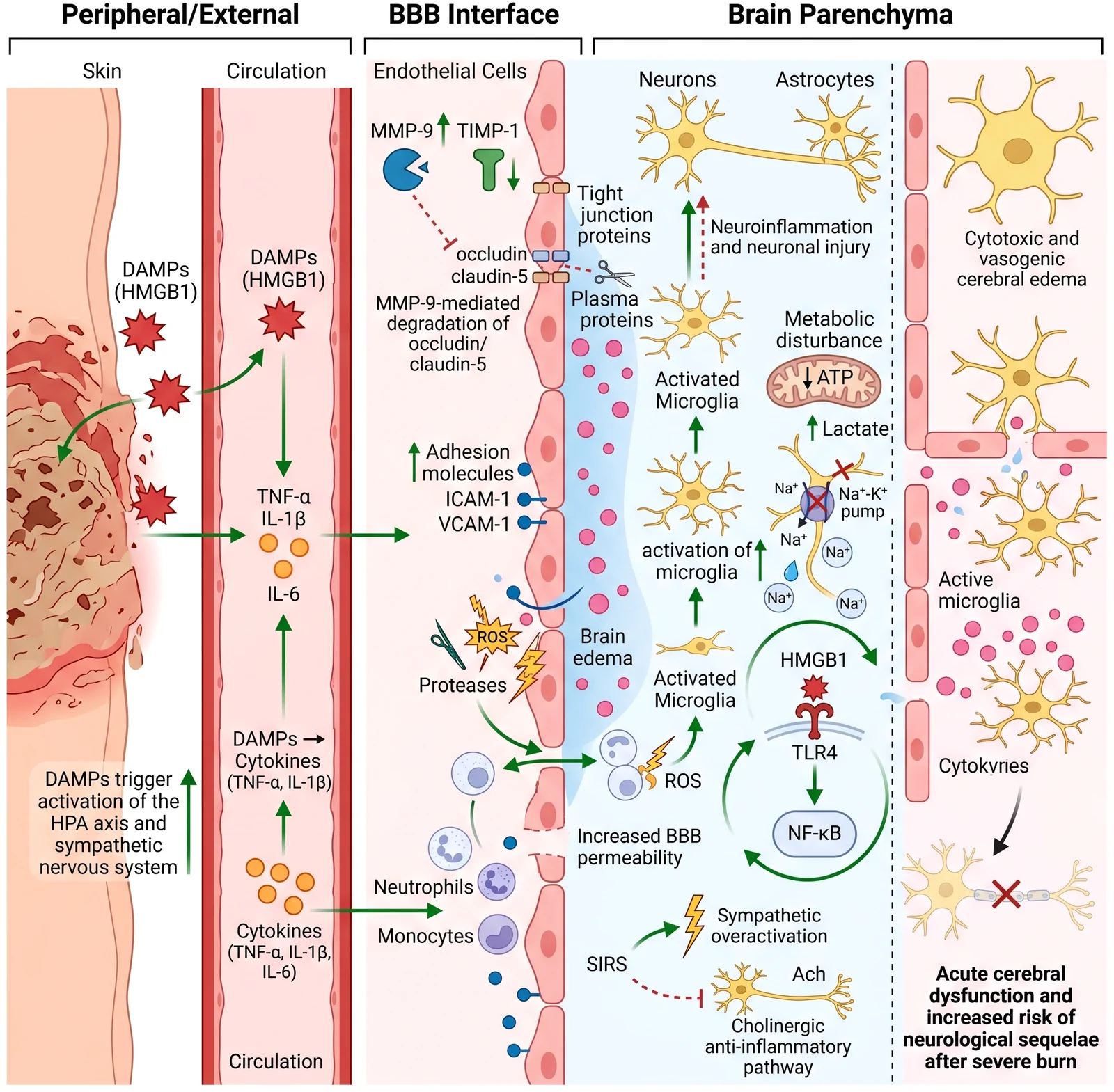
Mechanisms of traumatic brain injury caused by burns. Severe cutaneous burn releases DAMPs (e.g., HMGB1) into peripheral circulation. Circulating DAMPs and pro-inflammatory cytokines (TNF-*α*, IL-1*β*, IL-6) activate the HPA axis and sympathetic nervous system, triggering SIRS. At the BBB interface, ICAM-1 and VCAM-1 recruit neutrophils and monocytes. Elevated MMP-9 degrades tight junction proteins (occludin, claudin-5), while reduced TIMP-1 exacerbates endothelial barrier disruption, accompanied by ROS overproduction. Increased BBB permeability permits peripheral inflammatory factors to infiltrate the brain parenchyma, activating resident microglia. Activated microglia secrete ROS and HMGB1, which further activate TLR4/NF-*κ*B signaling to amplify neuroinflammation. This cascade impairs neuronal Na^+^/K^+^-ATPase activity, reduces ATP production, accumulates lactate, and drives cytotoxic/vasogenic cerebral edema. Sympathetic overactivation counteracts the cholinergic anti-inflammatory pathway, aggravating neuronal damage and raising the risk of long-term neurological sequelae after burn injury.

### 3.2 Cardiac injury

Myocardial injury caused by severe burns is a major cause of patient mortality and poor long-term prognosis (184). Myocardial injury caused by severe burns is one of the leading causes of death and poor long-term prognosis in patients. Acute myocardial injury can occur within 1 hour of severe burns, and early cardiac dysfunction is significantly associated with length of hospital stay and mortality (185). Cardiac injury in the early stages of burns is regulated by aseptic inflammation triggered by DAMPs, which activate innate immune pathways, leading to a surge in catecholamines, myocardial calcium overload, mitochondrial damage, and cardiomyocyte apoptosis (186). Aseptic inflammation releases substantial quantities of inflammatory mediators, which inhibit myocardial contraction, leading to a decrease in left ventricular ejection fraction, reduced cardiac output, and hypotension, all of which may precipitate acute heart failure (187).

The subacute phase begins 3 days to several weeks after injury, and eschar formation facilitates bacterial colonization, and PAMPs contribute to the immune response, leading to mixed inflammation (188). PAMPs activate signaling pathways, prompting cardiomyocytes to produce inflammatory mediators, which form the basis of sepsis-associated cardiomyopathy (189). Hypermetabolism brings about tachycardia, arrhythmias, deteriorated cardiac performance and abnormal myocardial markers, with cardiac vulnerability sustained over months or multiple years (112, 176).

A study by Duke et al. (190) Found that middle-aged and elderly patients with burns experienced nearly a threefold increase in hospital stays due to circulatory system diseases (IRR = 2.90) and a significant rise in long-term mortality (MRR = 1.11), indicating that burn-induced cardiovascular damage is a chronic pathological process that persists for decades and increases the risk of long-term mortality. Previous studies have shown that the incidence of cardiac complications 5–10 years after burns is 20%–30%. Patients with myocardial fibrosis have a poorer prognosis, with a 2- to 3-fold increased risk of heart failure and sudden cardiac death (191). The pathophysiological mechanisms primarily involve persistent neurohumoral activation, chronic oxidative stress, and myocardial remodeling and fibrosis (192). Recurrent IRI and mitochondrial dysfunction lead to myocardial energy deficiency, activating the TGF-*β*/Smad pathway, which promotes fibroblast proliferation and collagen deposition, resulting in myocardial fibrosis and impaired diastolic function (40). Persistent activation of the renin-angiotensin-aldosterone system (RAAS) and low-grade chronic inflammation exacerbate interstitial remodeling and reduce cardiac reserve (193). Patients commonly present with latent cardiac dysfunction, recurrent atrial fibrillation, or overt heart failure (194). Chronic cardiac injury affects organs such as the lungs and kidneys through neurohumoral regulation and inflammatory mediators, exacerbating systemic metabolic disorders and immunosuppression (192). In summary, early prevention and control of acute myocardial inflammatory injury and long-term fibrotic remodeling following burns are crucial for improving patient survival rates and quality of life.

### 3.3 Kidney injury

AKI after burns is a functional clinical syndrome with distinct temporal patterns (195). Studies have shown that the incidence of AKI in burn patients ranges from 30% to 50%, with an incidence of 29.4% for early-onset AKI within 72 hours of the burn and a mortality rate as high as 25.7% (196).

The core pathophysiological mechanism of early AKI lies in the kidney’s high sensitivity to ischemia and hypoxia. Burns, as a severe stressor, lead to excessive activation of the sympathetic nervous system and the renin-angiotensin system (197), resulting in severe renal vasoconstriction that reduces renal blood flow by more than 50% within 24 hours of injury (198, 199). A sharp decrease in effective circulating blood volume and inadequate renal perfusion are key factors leading to early AKI.

Extensive tissue necrosis at the burn site, combined with burn shock and IRI, leads to the release of large amounts of DAMPs into the systemic circulation, activating the innate immune cascade. This results in the release of large amounts of pro-inflammatory cytokines (200). In the kidneys, increased inflammatory cell infiltration and enhanced M1 polarization of macrophages can be observed, and systemic inflammatory signals can further spread to distant organs (201). Furthermore, extensive burns and inhalation injury are independent risk factors, and the mortality risk is several-fold higher compared to patients without early AKI (195). In addition to hemodynamic disturbances, rhabdomyolysis, pigment toxicity caused by hemolysis, resuscitation-related factors, and exposure to exogenous nephrotoxic drugs are also important pathogenic factors (202). These multiple insults lead to brush border detachment, mitochondrial swelling, and vacuolar degeneration of proximal tubular epithelial cells, which subsequently progress to acute tubular necrosis (ATN) accompanied by inflammatory cell infiltration (200). Persistent renal ischemia and hypoxia induce mitochondrial dysfunction, and the simultaneous activation of NADPH oxidase promotes the massive accumulation of ROS (203, 204). Excessive ROS damages lipids, proteins, and genetic material. The continuous depletion of endogenous antioxidants such as hyper-oxide (SOD) and glutathione leads to endoplasmic reticulum stress (205), which further induces brush border detachment, vacuolar degeneration, and mitochondrial swelling and necrosis in renal tubular epithelial cells. Ischemia and hypoxia simultaneously activate dual apoptotic pathways (206, 207). Previous studies have confirmed that the plasma of burn patients exerts a direct pro-apoptotic effect on renal tubular epithelial cells and podocytes, leading to changes in the permeability of these cells. This provides experimental evidence for the presence of nephrotoxic factors in the circulation in the context of burn injury (208). An imbalance in Bax/Bcl-2 expression in the mitochondrial pathway promotes the release of cytochrome c and activates proteins of the caspase family (209). At the same time, the Fas/FasL system of the death receptor pathway is activated, further amplifying apoptotic signals (157). Furthermore, impaired clearance of apoptotic bodies exacerbates the local inflammatory response (158). Abdominal compartment syndrome resulting from excessive resuscitation, along with hypotension induced by sedative-analgesic drugs, further reduces renal perfusion and amplifies various types of injury. Animal studies have confirmed that early peritoneal resuscitation can downregulate hypoxia-inducible factor 1-alpha (HIF1α) and NADPH oxidase 4 (NOX4), improve renal perfusion, and increase 24-hour survival rates (197, 210). Clinically, AKI at this stage is primarily characterized by a sudden drop in glomerular filtration rate (GFR), azotemia, and oliguria. Studies have shown that lactate dehydrogenase (LDH) levels within 24 hours of admission, in combination with the ABSI score, can predict the progression of injury early on (AUC 0.925), suggesting that early dynamic monitoring is crucial for assessing prognosis (202, 211).

As the disease progresses, the inflammatory and hypermetabolic states persisting for months post-burn, combined with recurrent infections and immune dysregulation, often precipitate renal tubular epithelial cell (TEC) damage and impaired repair, causing acute kidney injury to gradually progress to chronic kidney injury, particularly in the elderly (212).

Late-stage AKI accounts for approximately 10% of all cases and typically manifests during the subacute or chronic phases of burn recovery. Its pathophysiology is primarily attributed to infection, sepsis, septic shock, antimicrobial toxicity, a persistent inflammatory response, endothelial and microcirculatory dysfunction, and multi-organ dysfunction (196). The mechanisms of injury at this stage are complex. Specifically, the cytokine storm mediated by SIRS readily induces apoptosis in renal tubular cells and disrupts vascular permeability (201, 213). Membrane attack complexes formed by abnormal activation of the complement system further damage the glomerular filtration barrier (214). Oxidative stress and ferroptosis occupy vital positions within this process, and over-generated ROS consume endogenous antioxidant systems including SOD and GSH, while iron metabolism imbalance catalyzes lipid peroxidation via the Fenton reaction. Combined with reduced GPX4 activity, these factors collectively drive programmed cell death (203, 215). Furthermore, the mitochondrial apoptotic pathway mediated by Bax/Bcl-2 imbalance, the Fas/FasL death receptor pathway, and dysregulation of autophagy homeostasis synergistically exacerbate structural damage to the renal parenchyma (157, 215). Late-stage AKI often follows sepsis, with a mortality rate as high as 56.3% significantly higher than that of early-stage AKI and is more likely to drive progression from chronic kidney disease (CKD) to end-stage renal disease (ESRD) (196, 216). Given its role in exacerbating systemic multi-organ dysfunction and the increased difficulty of treatment, clinical management requires strict optimization of fluid resuscitation strategies and avoidance of nephrotoxic factors (216).

Recovery of renal function following burn-induced AKI is often incomplete. Even when clinical indicators return to normal, latent renal parenchymal damage may persist.

Cohort studies have confirmed that AKI survivors treated with RRT often present with persistent albuminuria, hypertension, and metabolic disorders, and have a significantly increased long-term risk of developing CKD, with an even higher risk among those who received RRT (217). According to long-term follow-up data, 91% of burn-injured AKI subjects treated with CRRT recovered their baseline kidney function in 3-year follow-up and long-term-dialysis dependency occurred in only 18%, with their prognosis superior to ordinary ICU-admitted AKI patients (218). Long-term latent renal damage includes tubulointerstitial fibrosis, glomerulosclerosis, and renal vascular remodeling, driven by residual chronic inflammation, oxidative stress, drug toxicity, and metabolic abnormalities (219). Clinically, stratified follow-up should be implemented. All AKI survivors should undergo annual testing of eGFR, urinary microalbumin-to-creatinine ratio, KIM-1, and NGAL. Patients at high risk for RRT, sepsis, or multiple surgeries should undergo follow-up examinations every 3–6 months and undergo a renal ultrasound (219). Among them, patients diagnosed with CKD should receive early treatment with ACEIs/ARBs and undergo strict control of blood pressure, blood glucose, and blood lipids (200). Subsequent multicenter cohort studies are needed to identify genetic, inflammatory, and metabolic biomarkers associated with the progression of burn-related AKI to CKD, establish a personalized follow-up system, and investigate the efficacy of SGLT2 inhibitors and endothelin receptor antagonists in the management of chronic kidney injury (220).

### 3.4 Lung injury

Due to the unique physiological structure of the lungs, the upper respiratory tract is highly susceptible to direct thermal injury following burns, while the lower respiratory tract and lung parenchyma are often damaged by the inhalation of toxic gases and particulate matter. Inhalation injury is one of the strongest independent predictors of ARDS, pneumonia, prolonged mechanical ventilation, sepsis, and death following severe burns (221).

Epidemiological data indicate the ARDS incidence reaches 24% among burn patients complicated by inhalation injury, and the case-fatality rate can exceed 31% after ARDS onset. This mortality figure is markedly higher than that of burn victims free from pulmonary injury, rendering inhalation-related lung damage the leading independent risk factor for early post-burn death (222).

In the early stages, thermal energy and chemicals in smoke directly damage the airway mucosa, triggering a severe aseptic inflammatory response. Specifically, carbon monoxide impairs oxygen delivery by forming carboxyhemoglobin and interfering with mitochondrial respiration, accounting for approximately 80% of fire-related deaths (223). Cyanide suppresses cytochrome c oxidase and further disrupts cellular oxygen utilization, and raised cyanide concentrations are commonly identified among smoke-inhalation patients (224). These early events trigger local and systemic aseptic inflammatory responses, leading to an exuberant release of pro-inflammatory cytokines and damage-associated molecular patterns, which subsequently activate innate immune signaling pathways. These pathways function to detect danger signals and amplify inflammation, thereby promoting the infiltration of neutrophils into lung tissue (225). Common manifestations include congestion, edema, and necrosis of the airway mucosa (226). This inflammatory response disrupts the barrier function of the airway epithelium, leading to impaired ciliary clearance and suppression of local immune function, thereby creating conditions conducive to bacterial colonization and infection (227).

As the condition progresses, impaired ciliary clearance in the airway mucosa, reduced phagocytic capacity of alveolar macrophages, and local immunosuppression set in, transforming what was originally a sterile inflammatory focus into a nidus for bacterial colonization and proliferation (228). Post-burn bacterial translocation from the gut and the spread of pathogens from infected skin wounds via the bloodstream or the airways further increase the risk of pulmonary infection, driving the disease process from aseptic inflammation toward infectious pneumonia and even sepsis (229).

Prognostically, the impact of inhalation injury on patients with severe burns often exceeds that of the extent of the burn itself (230). Recent cohort studies have demonstrated that burn patients with concomitant inhalation injury have a significantly higher risk of ARDS, a greater requirement for prolonged mechanical ventilation, and an increased risk of mortality. Moreover, mortality rates increase in a stepwise manner with the severity of inhalation injury (231). Therefore, as the disease progresses, inhalation injury is not merely a localized airway injury but also a key factor in the gradual progression of early aseptic pulmonary inflammation following burns to late-stage systemic infection and even multi-organ failure (232). Consequently, early and aggressive airway management, antimicrobial therapy, and supportive care are of critical importance (233).

### 3.5 Liver damage

Following severe burns, dysfunction of the liver—a key organ in the body’s metabolism and immune regulation—occurs in as many as 30% to 50% of cases and is closely associated with patient prognosis (234). As the core organ of human metabolism, the liver exhibits complex and diverse mechanisms of injury (235), mainly involving oxidative stress, inflammatory responses, apoptosis, microcirculatory disturbances, and metabolic disorders. These processes are often accompanied by a series of characteristic morphological changes in liver tissue (236).

Early organ injury following burns is primarily mediated by sterile inflammation triggered by endogenous DAMPs (237). Extensive tissue necrosis at the burn wound site, compounded by burn shock and IRI, leads to the massive release of DAMPs into the bloodstream, thereby activating the innate immune cascade (238). The NLRP3/caspase-1/GSDMD axis triggers pyroptosis, while the RIPK1/RIPK3/MLKL signaling pathway initiates necroptosis. Inhibition of GPX4 by ROS and free Fe²^+^ leads to lipid peroxidation and subsequently triggers ferroptosis in hepatocytes and sinusoidal endothelial cells (239, 240). Ferroptosis-induced damage to sinusoidal endothelial cells accelerates the formation of intrahepatic microthrombi and exacerbates hypoxia, further worsening the clinical condition. Following clinical resuscitation and restoration of hepatic perfusion, IRI further aggravates liver tissue damage, while mitochondrial dysfunction and endoplasmic reticulum stress-mediated calcium overload continue to amplify oxidative stress levels (241). On days 3 to 5 post-injury, PAMPs originating from wound and pulmonary infections activate Kupffer cells and sinusoidal endothelial cells via pathways such as Toll-like receptors, further exacerbating liver injury (242). The sustained activation of Kupffer cells, impaired microcirculation in the hepatic sinusoids, and recurrent inflammatory cell infiltration collectively drive the progression from protracted liver injury to chronic liver injury (243).

The early clinical manifestations of liver injury following burns are often subtle and primarily include symptoms such as fatigue, anorexia, nausea, and abdominal distension, which are easily masked by burn pain and the stress response (234). Jaundice, on the other hand, tends to become more pronounced in the later stages (244). Therefore, clinicians rely more heavily on dynamic changes in laboratory parameters for identification. Laboratory parameters exhibit distinct temporal changes. Within 24 hours of a burn injury, a sharp rise in the liver cell damage markers transaminases (AST/ALT) can be observed. Animal studies suggest that hepatic edema and the release of enzymatic markers may occur as early as 12 hours after injury (245). Early liver function abnormalities often indicate the onset of multi-organ dysfunction syndrome and a poor prognosis (246).

Unlike in the early stages of burns, late-stage liver injury is closely associated with wound and pulmonary infections, inflammation cascades mediated by pathogen-associated molecular patterns, and sepsis. This symptom typically occurs within a few days of injury and is more common in hosts with persistent immunosuppression. This symptom typically appears within a few days of injury and is more common in hosts with persistent immunosuppression (247). The factors determining whether this transition occurs are multidimensional, with total burn area and depth serving as foundational variables (248). The quality of fluid resuscitation directly influences the severity of IRI and intestinal barrier function (249). The timeliness and thoroughness of wound management are core clinical interventions for reducing bacterial colonization and delaying or preventing this phase transition (250). The host’s baseline immune function, nutritional status, and concomitant inhalation injuries also significantly influence the timing and severity of this transition (251).

Furthermore, extensive hepatocyte necrosis during the acute phase continuously impairs the liver’s synthetic, detoxification, and metabolic functions, ultimately progressing to prolonged post-burn liver dysfunction. This condition is prone to inducing various types of systemic metabolic complications, including glucose metabolism disorders, lipid metabolism disorders, hypoalbuminemia, and decreased detoxification function. Circulating toxic substances originating from the liver can damage distal organs via the bloodstream. In severe cases, this may lead to the onset and progression of MODS (252). The core driving factors are persistent activation of Kupffer cells, impaired microcirculation in the hepatic sinusoids, and recurrent inflammatory cell infiltration (242).

As the condition progresses, laboratory test results in burn patients show rising levels of bilirubin and markers associated with burn-associated cholestasis (BAC), indicating impaired bile production or excretion (253). Burn-associated cholestasis typically occurs in the middle to late stages of the disease course, occurring at an average of 11 days post-injury (253). In the late stages of burns, decreased albumin levels and impaired hepatic synthetic function can be observed, indicating an increased risk of poor prognosis (254). Imaging studies and autopsy findings have shown that patients with severe burns also exhibit visible hepatocyte edema, steatosis, and necrosis in the late stages (255). Animal studies have also confirmed these histopathological changes (256).

In summary, it is essential to assess the overall nutritional and metabolic status of burn patients even after discharge and to conduct long-term, dynamic monitoring of liver function biochemical markers to identify hepatic complications as early as possible. See **Figure 4** for details (257).

**Figure 4:**
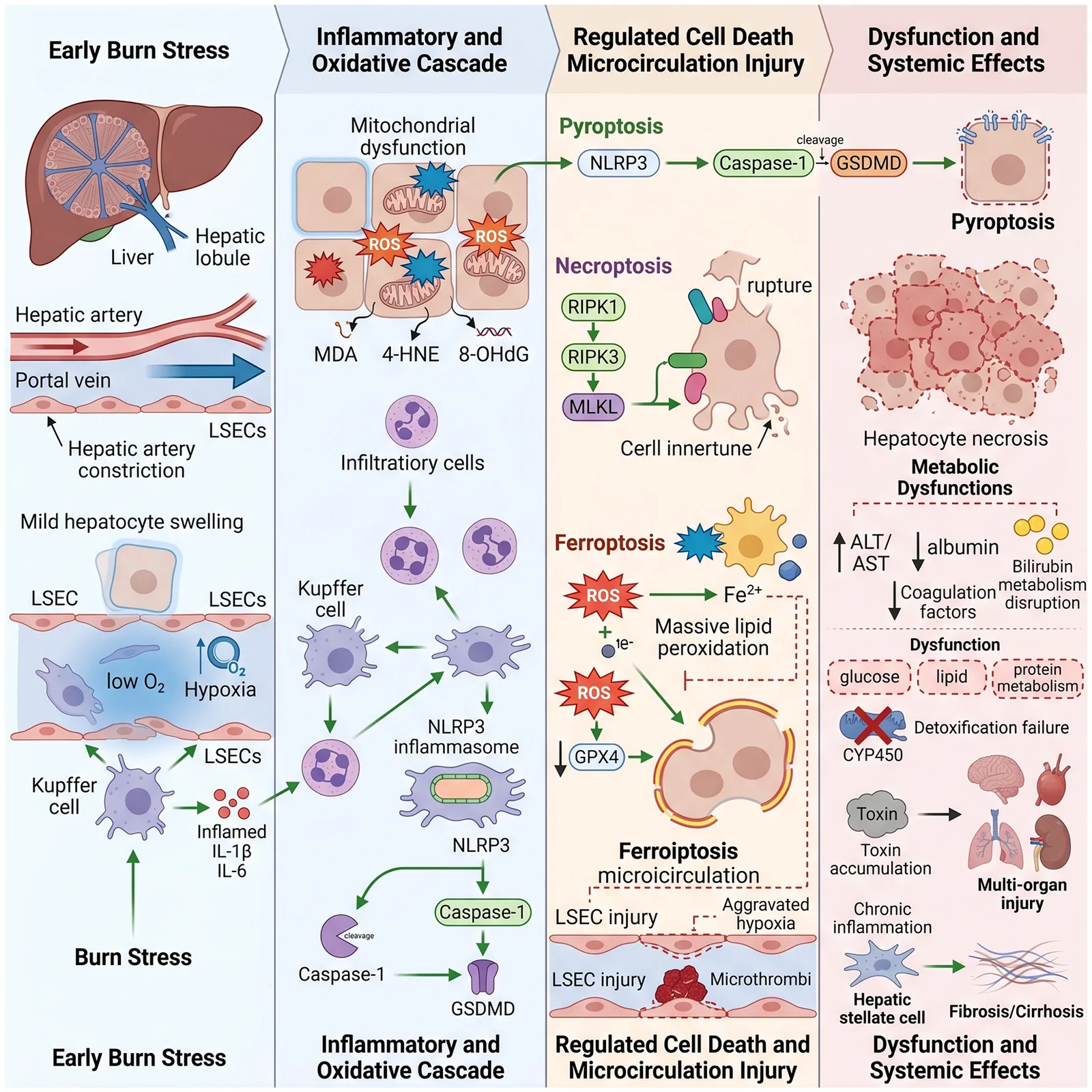
Mechanisms of liver injury following burns. (1) Early burn stress: Burn injury induces hepatic vasoconstriction, lobular hypoxia and liver sinusoidal endothelial cell (LSEC)swelling, which activates Kupffer cells to secrete IL-1*β* and IL-6. (2) Oxidative and inflammatory cascade: Hypoxia and inflammation disrupt mitochondria and boost ROS, increasing MDA,4-HNE and 8-OHdG.Oxidative stress further activates Kupffer cell NLRP3 inflammasome, driving caspase-1-mediated GSDMD cleavage. (3) Regulated cell death and microcirculatory injury: The NLRP3/caspase-1/GSDMD axis triggers pyroptosis, whereas RIPK1/RIPK3/MLKL signaling initiates necroptosis. ROS and free Fe²^+^ suppress GPX4 to drive lipid peroxidation and ferroptosis in hepatocytes and LSECs. Ferroptotic LSEC damage accelerates intrahepatic microthrombosis and worsens hypoxia. (4) Hepatic failure and systemic complications: Combined pyroptosis, necroptosis and ferroptosis trigger widespread hepatocyte necrosis, accompanied by elevated ALT/AST, hypoalbuminemia, impaired bilirubin and coagulation metabolism, and compromised CYP450 detoxification. Chronic inflammation activates hepatic stellate cells to drive liver fibrosis/cirrhosis and subsequent multi-organ injury.

### 3.6 Gastrointestinal injury

Among critically ill burn patients, the incidence of gastrointestinal motility disorders is 51%, and these disorders typically occur approximately 32 hours after admission to the ICU, which is earlier than in the general ICU population (258). In another Chinese cohort study of severe burn patients, the incidence of gastrointestinal dysfunction was 45.4%, and it was associated with a variety of clinical manifestations (259). The pathophysiological mechanisms underlying gastrointestinal injury following burns remain a subject of intense investigation. Specifically, a sudden drop in effective circulating blood volume triggers visceral vasoconstriction, leading to inadequate intestinal perfusion and IRI (260). This process is accompanied by increased capillary permeability and microthrombus formation (63). The main characteristics of the acute phase include gastrointestinal dysfunction and peristaltic disorders, as well as stress ulcers and bleeding, intestinal paralysis, and food intolerance (259). The stress response activates the sympathetic nervous system, promoting the massive release of catecholamines, which causes blood flow redistribution and further exacerbates mucosal ischemia and hypoxia (261). Concurrently, DAMPs trigger an innate immune response, induce a cytokine storm and disrupt the tight junctions of the intestinal epithelium (53, 262). Combined exposure to alcohol or radiation can synergistically downregulate REG3G expression and inhibit mucin synthesis, significantly exacerbating barrier dysfunction (263). Clinical manifestations include stress ulcers, gastrointestinal bleeding, and intestinal perforation. In some cases, the condition progresses to multi-organ dysfunction (264). Epidemiological data show that patients with concomitant gastrointestinal injury have a significantly increased risk of inhalation injury and infectious complications, and these complications are important predictors of poor prognosis in critically ill burn patients (264, 265).

The persistent inflammatory response and neuroendocrine dysregulation induced by burns underlie long-term gastrointestinal damage (266). An imbalance between pro-inflammatory and anti-inflammatory factors leads to gut dysbiosis and bacterial translocation. Bacteria and their lipopolysaccharides activate immune responses in distal organs via the portal venous system, thereby mediating systemic inflammatory response syndrome and secondary liver injury (267).

The overall burden of gastrointestinal complications is high among patients with severe burns (268). Long-term follow-up studies have shown that the risk of hospitalization due to digestive system diseases remains high after burns. Both adults and children experience an increase in the number of hospitalizations and the length of hospital stays caused by digestive system diseases. This risk can persist for up to 20 years (269). This provides evidence supporting the view that the effects of burns on patients persist for a long time after the initial injury.

Dysfunction of the vagus nerve-mediated gut-brain axis directly influences the regulation of the body’s stress response. If this dysfunction persists, it will exacerbate the onset and progression of multi-organ dysfunction syndrome (270, 271). Disruption of the interactions between the gut, the microbiota, and distal organs can mediate damage to distal organs through various signaling pathways, which is a key underlying mechanism of post-burn multi-organ dysfunction syndrome (271).

Overall, the core therapeutic interventions for gastrointestinal injury following severe burns focus on correcting hemodynamic disturbances, improving microcirculatory dysfunction, inhibiting inflammatory cascades, and restoring the body’s pro-inflammatory/anti-inflammatory balance.

Long-term gastrointestinal damage is primarily characterized by persistent impairment of digestive and absorptive functions and motility abnormalities. Patients suffer from alternating intractable diarrhea and constipation, steatorrhea, protein-losing enteropathy and trace-element deficiencies, and such manifestations may last for months and greatly hinder nutritional rehabilitation (259). Among patients with severe burns, the incidence of persistent gastrointestinal dysfunction is approximately 45.4%, and these symptoms can persist for up to one year after the burn (272). Prognostic assessments indicate that the degree of increased intestinal permeability measured in the second week after burns is independently associated with the subsequent incidence of sepsis and 90-day mortality, after adjusting for burn area and age (259, 273). Intestinal barrier function indicators are increasingly acknowledged as critical adjunctive parameters within burn prognostic frameworks, distinct from conventional organ failure scores, and their influence on clinical decision-making is undergoing re-evaluation (273).

### 3.7 Islet damage

Severe burns induce systemic hypermetabolism and excessive neuroendocrine activation, prompting the massive release of catecholamines and cortisol, which leads to a dramatic increase in energy expenditure and accelerated protein catabolism (122). Animal studies in mice have shown that as early as 24 hours after severe burns, elevated fasting blood glucose levels and reduced glucose-stimulated insulin secretion can occur, accompanied by increased insulin-like growth factor-1 (IGF-1) levels in the pancreas, decreased activity of complex III of the mitochondrial respiratory chain, and downregulation of antioxidant enzyme expression (168). Separate mouse studies suggest that silenced information regulator 3 (SIRT3) downregulation is associated with mitochondrial oxidative stress and islet cell apoptosis (274). Acute islet injury in the early stages of burns is primarily characterized by impaired insulin secretion and peripheral insulin resistance, which directly lead to stress-induced hyperglycemia (275). The underlying mechanisms involve the synergistic interaction of multiple pathways, including excessive release of stress hormones, systemic inflammatory responses, oxidative stress, and mitochondrial dysfunction (276).

Specifically, epinephrine and other catecholamines activate *β*-adrenergic receptors. On the one hand, studies in both mice and humans following burns have observed that these catecholamines directly promote lipid hydrolysis in adipose tissue and the release of free fatty acids (111). On the other hand, researches based on heat-injured mouse models prove browning induction within white adipose tissue, and the two mechanisms together aggravate systemic metabolic disturbance and block insulin-signaling cascades (111, 123). Furthermore, inflammatory cytokines such as IL-6 also contribute to the development of insulin resistance (277). Clinically, hyperglycemia is closely associated with delayed wound healing, an increased risk of infection, and prolonged hospital stays (278). Epidemiological studies show that the incidence of stress-induced hyperglycemia in patients with severe burns can reach 30%–50%, and hyperglycemia is an independent risk factor for early mortality (279).

In addition to the acute-phase response, the body remains in a state of high metabolism, high glucose levels, and high energy expenditure for an extended period following a burn injury (111). A real-world cohort study based on TriNetX (adults, ≥30% TBSA, no prior history of diabetes, propensity score-matched) showed that the 15-year cumulative incidence of diabetes was 16.5% in the burn group and 12.2% in the control group(*log-rank P* = 0.0003), suggesting that severe burns are associated with an increased risk of long-term diabetes (280). A cohort study of children with burns found that children with a history of burns had elevated plasma levels of branched-chain and aromatic amino acids, metabolites that are associated with an increased risk of type 2 diabetes. The study also noted that the 5-year risk of developing type 2 diabetes among children with burns was approximately 2.21 times higher (281).

Impaired pancreatic function leads to persistent *β*-cell failure, which is the key factor underlying the long-term persistence of hyperglycemia after a burn injury (111). Mitochondrial dysfunction drives oxidative stress. Burns downregulate the activity of respiratory chain complex III and suppress the expression of antioxidant molecules such as Trx2 and Mn-SOD (275). Furthermore, reduced Sirt3 expression leads to abnormal deacetylation of Mn-SOD and IDH2, exacerbating mitochondrial damage, inducing apoptosis, and impairing glucose-stimulated insulin secretion (274). Furthermore, endoplasmic reticulum (ER) stress gets activated, with burns-triggered upregulation of the UPR markers BIP, p-PERK and CHOP (168), and sustained stress activates the caspase-3 apoptotic pathway, thereby inhibiting insulin secretion. It has been demonstrated that 4-PBA intervention improves glucose tolerance, confirming the central role of this pathway (168). At the signaling level, burns inhibit PI3K/Akt activity, promote FoxO1 nuclear translocation, and downregulate PDX-1 expression, leading to *β*-cell dysfunction (282), whereas activation of this pathway restores insulin secretion (283). These long-term damages lead some patients to develop persistent impaired glucose tolerance or new-onset diabetes, and may result in microvascular complications, with retinal, renal, and neuropathic complications being the most common (284). They also increase the risk of adverse cardiovascular events and impair multiorgan function. Epidemiological studies indicate that the incidence of diabetes in burn survivors is 2–3 times higher than in the general population over the long term (280). Impaired pancreatic function acts as an independent risk factor for long-term all-cause mortality and cardiovascular events, so clinicians should attach great importance to this indicator for prognostic evaluation during comprehensive patient management. Current research has shifted toward network mechanisms involving cross-talk among multiple pathways, with a focus on identifying core targets (285, 286).

### 3.8 Musculoskeletal injuries

During the acute burn phase, musculoskeletal injuries are common. Severe burns (>30% TBSA) trigger an immediate and sustained hypermetabolic response during the acute phase, characterized by rapid skeletal muscle wasting (287). Muscle protein turnover shifts dramatically toward net degradation within days of injury, contributing to significant morbidity and prolonged recovery (126). A prospective ultrasound study found that quadriceps thickness in hospitalized burn patients decreased from days 1 to 7, with an average reduction of about 4.4–4.95 mm, and significant changes were seen between days 1 and 3 (287). High temperatures directly cause protein denaturation, cell necrosis, and muscle fiber damage. Articular cartilage is highly susceptible to heat-induced degradation. Inflammatory factors and glucocorticoids promote bone resorption (288, 289). Inflammatory factors and glucocorticoids promote bone resorption by upregulating the RANKL pathway, which fosters osteoclast differentiation and increases bone resorption during the acute phase (290). Tissue ischemia and hypoxia from microcirculatory disorders impair muscle energy metabolism, exacerbating ATP depletion and lactate accumulation, leading to rhabdomyolysis (291). Clinical features include acute bone loss, muscle weakness, myalgia, and elevated creatine kinase, while severe presentations can lead to acute tubular necrosis (292). Notably, muscle atrophy is asymmetric, affecting trunk core and proximal lower limb muscles most (287). Bed rest, immobilization, and delayed rehabilitation all exacerbate disuse atrophy and joint stiffness (293). Randomized trials show that starting resistance and aerobic training about 7 days post-admission is safe for severe burn patients, reducing quadriceps loss and aiding strength recovery. Therefore, healthcare professionals should initiate rehabilitation as early as possible. Early intervention can also be achieved during the intensive care phase through passive movement, position management, and the use of orthoses. The long-term effects of burns on skeletal muscle bioenergetics remain unclear. Impaired mitochondrial function can persist for up to two years after discharge, leading to ongoing physiological stress (294). Chronic high metabolism in burn patients underlies lasting injury, with joint dysfunction at discharge in 22%–42% and heterotopic ossification in 0.2%–4%, though severe burns can increase these rates to over 50% (290, 295). Studies on pediatric burn patients further confirm that children with extensive burns experience more significant impairments in growth, development, and motor function, suggesting a heavier burden on their skeletal muscles and motor outcomes (296). The mechanisms of chronic-phase damage primarily involve long-term neuroendocrine dysregulation. Over the course of months to years following a burn, persistently elevated levels of catecholamines and cortisol increase resting energy expenditure (297), activate the ubiquitin-proteasome pathway to promote myofibrillar protein degradation, and induce severe skeletal muscle atrophy (298). Concurrently, chronic low-grade inflammation and an imbalance in MMP-9/TIMP-1 can lead to damage to distal tissues and organs as well as heterotopic ossification (299). Mitochondrial dysfunction lowers ATP and raises ROS, damaging muscle fibers through oxidative stress and triggering apoptosis (300). Poor microcirculatory perfusion worsens ischemic damage (301). Oxidative stress damages muscle and mitochondrial structures, disrupts bone cell balance, and accelerates cartilage damage (302). Inflammatory factors, glucocorticoids and oxidative-stress trigger osteoclast activation and suppress osteogenesis, and coupled with vitamin D deficiency and disruption of the GH/IGF-1 axis, these changes raise the risks of osteoporosis and bone fracture (303). Abnormal collagen deposition and muscle remodeling via the TGF-*β*1/Smad2/3 pathway are key mechanisms of joint contractures (304). Long-term complications mainly involve muscle weakness and joint contractures, impairing metabolism and respiratory function, increasing cardiopulmonary workload, and forming a vicious cycle (40). Clinical studies show 30% to 50% of severe burn patients have persistent joint dysfunction within a year, linked to chronic fatigue, infection risk, and lower quality of life (305, 306). Musculoskeletal injury severity is now part of the MODS scoring system as a predictor of prognosis (307). Early identification and effective rehabilitation are crucial for improving burn patients’ quality of life.

## 4 Management strategies for severe burns resulting in damage to vital organs

### 4.1 Early fluid resuscitation and circulatory support

Severe burns trigger systemic inflammation and capillary leak, reducing blood volume and worsening organ damage. Early fluid resuscitation and circulatory support are crucial to prevent organ failure. These measures stem from burn-induced circulatory dysfunction. Inflammatory mediators initiate multi-organ injury (308), with circulatory dysfunction as the core issue. DAMPs increase capillary permeability (309, 310), while factors like dipeptidyl peptidase 3 (DPP3) suppress cardiac contractility, linking stroke volume to prognosis (311). In critical illness, the lungs and kidneys are vulnerable, and biomarkers like proenkephalin (proenkephalin A 119-159, penKid) can signal AKI risk (312).

Advanced fluid resuscitation for burn patients has shifted from empirical formulas (e.g., Parkland and Brooke) to a personalized, goal-directed model. Traditional formulas guide early treatment but often cause under- or over-resuscitation by ignoring individual differences. Hourly urine output monitoring is a key endpoint, though it is affected by prerenal, renal, and postrenal factors. Decreased urine output within 12 hours post-injury suggests hypovolemia, but inflammation and ischemia later impact it. Clinicians must monitor closely for fluid creep and its consequences (313). Urine output alone is insufficient, hence comprehensive assessment is needed (202). Meta-analyses show a survival benefit in observational studies, but not in RCTs. Well-designed multicenter RCTs are required to compare hemodynamic-guided resuscitation with traditional approaches.

The standard clinical principle for fluid resuscitation in burn patients uses crystalloids as primary replacement, supplemented by colloids. For extensive burns, albumin limits resuscitation volume (314). Its use is controversial, especially regarding timing (314). Albumin helps maintain plasma colloid osmotic pressure, yet it may extravasate into the interstitial space during the early post-burn phase, potentially exacerbating edema. Current guidelines advise avoiding albumin within the first 24 hours, though it may be considered later for hypoproteinemia or poor crystalloid response (315). Albumin reduces fluid input in the first 48 hours, but strong evidence for reducing acute kidney injury or promoting wound healing is lacking (314).

Strict monitoring of electrolyte disturbance is necessary when using hypertonic salt solution, and its long-term clinical benefits require further verification (308). The organ protection potential of new resuscitation fluids is under investigation. Animal studies utilizing severely burned rat models demonstrate that a low-dose resuscitation regimen comprising adenosine, lidocaine, and magnesium (ALM) mitigates oxidative stress-induced lung tissue damage and preserves alveolar structural integrity (316).

Advanced circulation monitoring and organ protection are needed for comprehensive treatment. Technologies like PiCCO-guided guide individualized plans. If low mean arterial pressure persists despite fluids, or if cardiac insufficiency exists, vasoactive drugs may be used (317, 318). Lung protection includes restrictive fluid management and ventilation. Refractory hypoxemia requires early prone positioning or ECMO (319). Renal protection involves avoiding nephrotoxic drugs, and AKI patients need early CRRT (320). Cardiac and cerebral protection focuses on stable perfusion, heart rate control, and reducing intracranial pressure. Severe burns can cause cerebral edema and encephalopathy (321).

The fluid resuscitation paradigm for burn patients faces challenges such as insufficient precision, inconsistent endpoint criteria, and lack of evidence-based support for adjunctive therapies (322). Future research should focus on individualized protocols under multimodal monitoring and validate biomarkers like DPP3 and penKid (312, 323). Adjunctive therapies targeting inflammation and endothelial injury should reduce fluid volume (316, 324). Clinicians must shift to a precise balance, combining multi-organ support and targeted adjuvant therapy to lower multi-organ failure risk.

### 4.2 Systemic immune modulation and comprehensive strategies for the prevention and treatment of infections

#### 4.2.1 Early surgical debridement and wound closure

Early and thorough debridement of necrotic tissue and wound closure are the most direct and effective interventions for eliminating the triggers of uncontrolled inflammation, hypermetabolic states, and sepsis following severe burns (325). The widespread adoption of this clinical practice has historically correlated with a significant decline in burn-related mortality, marking a landmark transformation in the field of burn treatment (326). Surgical debridement directly eliminates the source of necrotic substances that continuously activate the inflammatory response by removing devitalized tissue, thereby effectively curbing the vicious cycle of systemic inflammatory response syndrome (327).

Furthermore, timely wound coverage restores the skin’s barrier function, mitigating water evaporation and heat loss while preventing the invasion of pathogenic microorganisms, and creates favorable conditions for subsequent metabolic support and organ function recovery (328). Patients with extensive burns (exceeding 50% of the body surface area) accompanied by inhalation injuries have an extremely high mortality rate, while early surgical intervention is crucial for controlling systemic inflammatory response and preventing the onset of MODS (329).

However, the decision to perform debridement requires careful consideration of multiple factors. Blood loss associated with extensive debridement may exceed anticipated levels, so the patient’s hemodynamic compensatory capacity must be carefully assessed, and blood transfusion should be administered when necessary (330). The cumulative physiological toll of multiple surgeries must also not be overlooked, including anesthetic risks, exacerbated metabolic stress, and nutritional depletion. In particular, perioperative anesthetic management requires multidisciplinary collaboration to address challenges such as fluid resuscitation, thermoregulation, and airway management (331). Therefore, the timing of surgery is particularly critical for burn patients with concomitant inhalation injuries or hemodynamic instability.

In summary, premature debridement may exacerbate systemic shock, while delayed debridement may result in missing the therapeutic window to interrupt the inflammatory cascade (332). Consequently, debridement strategies should be highly individualized by thoroughly evaluating factors such as the patient’s overall condition, wound depth, and risk of infection to develop targeted and effective interventions. In recent years, the application of minimally invasive techniques, such as enzymatic debridement, has provided new options for some patients, yet their exact role in severe burns requires further study (333).

#### 4.2.2 Infection control and optimization of antimicrobial therapy

Severe burns can cause systemic pathological and physiological disorders, thus causing secondary multi-organ injuries. At the same time, skin barrier defects and the combined effect of immune suppression will make patients face extremely high risk of invasive infection. Infection and organ injury are interrelated and easy to form a vicious circle. They are the core risk factors for multi-organ dysfunction syndrome after burn and death of patients (329, 334, 335).

The pathogen spectrum causing burn infection has obvious temporal dynamic changes. In the early stage of burn, wound infection is mainly caused by gram-positive cocci such as Staphylococcus aureus (334). As hospital stays lengthen, non-fermentative Gram-negative pathogens including Pseudomonas aeruginosa and Acinetobacter baumannii gradually dominate infectious isolates. Meanwhile, the incidence of invasive fungal infections, such as candidiasis and mold infections, rises markedly (336). Clinically, the detection frequency of multidrug-resistant and extensively drug-resistant strains remains persistently high. Pathogens such as methicillin-resistant Staphylococcus aureus (MRSA) and carbapenem-resistant bacterial isolates further complicate the selection and efficacy of anti-infective regimens (337, 338). Furthermore, wound surface biofilms mediate persistent and refractory infections, further increasing the burden of infection prevention and control (339). In terms of diagnostic systems, traditional pathogen culture and drug susceptibility testing remain the primary methods. Although significant scientific advances have been made with new technologies such as metagenomic sequencing and scanning electron microscopy, the translation of these findings into clinical practice still faces numerous constraints due to a lack of clinical trials in burn patients, inconsistent definitions, and systemic barriers to antimicrobial therapy (131, 340).

The core means of burn infection prevention and control is thorough wound debridement. Early excision and skin grafting, using antibacterial dressing covering, negative pressure treatment of wound surface and other methods can effectively remove the source of infection (329, 341). Given the compromised immune function of burn patients, they are highly susceptible to colonization by multidrug-resistant bacteria. In clinical environment, aseptic operation and contact isolation system must be strictly implemented, and drug-resistant bacteria screening should be actively carried out at the same time, avoid cross-transmission in the hospital (338). At the same time, organ protective cluster intervention measures should be implemented. Lung protective ventilation strategy can reduce the risk of pulmonary infection and lung injury (342). Kidney protection measures include avoiding the use of nephrotoxic drugs, accurately adjusting the dosage of antibiotics according to renal function, and initiating renal replacement therapy when necessary (343, 344). Intestinal protection mainly relies on early enteral nutrition and selective digestive decontamination, in order to reduce bacterial and endotoxin displacement, prevent intestinal infection (345, 346).

Antimicrobial Therapy follows the principles of stratification, individualization and accuracy. The initial empirical antibacterial plan needs to be formulated in combination with the infection risk of the patient and the pathogen epidemic situation in the ward, covering Gram-positive and gram-negative bacteria, focus on optimizing treatment strategies for high-risk groups vulnerable to multi-drug resistant bacteria (336, 347). Taking into account the specific pathophysiological changes and organ function status associated with burns, drug dosages, frequencies, and treatment durations should be dynamically adjusted through therapeutic drug monitoring to balance antimicrobial efficacy with organ toxicity and damage (343). For special infections such as fungal and viral infections, a screening system for high-risk populations should be established to implement targeted antimicrobial interventions (348). For infections caused by drug-resistant bacteria, combination regimens using classic older drugs such as polymyxin, tigecycline, and fosfomycin may be employed, or newer broad-spectrum antibiotics such as cefdirome may be selected (347). Concurrently, non-antibiotic strategies—including phage therapy, quorum sensing inhibition, and immune-modulating interventions—have emerged as novel adjunctive approaches for managing drug-resistant bacterial infections (339, 349, 350).

Future burn infection prevention and control systems will integrate microbiomics, host immune characteristics, and pharmacokinetic/pharmacodynamic models to establish a multimodal, precision-based anti-infection system. By leveraging a multidisciplinary collaborative model that combines wound management, precision antimicrobial therapy, organ function support, and cutting-edge biological therapies, this approach aims to improve long-term outcomes for patients with severe burns and reduce mortality rates (343).

#### 4.2.3 Immune modulation and targeted anti-inflammatory therapy

Inflammation and immune suppression after severe burns drive multi-organ injuries. Current immune regulation and anti-inflammatory therapy form a multi-level intervention system (351). The core approach is to block inflammatory cascades, reverse immune paralysis, and restore immune homeostasis to protect organs and improve prognosis (352).

In traditional anti-inflammatory regimens, NSAIDs mainly inhibit cyclooxygenase, but their limited target specificity and risk of kidney injury restrict them to auxiliary use (353). Glucocorticoids have strong non-specific activity, effective in early burn stages at low doses (354), but high doses may worsen immune inhibition and delay wound healing (355). Novel selective anti-inflammatory modulators are being developed to balance efficacy and safety (356). Targeted anti-inflammatory therapy, including cytokine antagonists like tocilizumab and anakinra, is a key focus (357, 358), though it requires biomarker-based patient stratification. Complement inhibitors (such as eculizumab) can block abnormal complement activation, and animal studies show they effectively reduce burn-induced lung and myocardial injury, with clinical translation ongoing (359, 360). Small-molecule inhibitors targeting pathways such as JAK/STAT and p38 MAPK block inflammatory signaling and offer oral administration, but their clinical safety needs further validation (361–363). Immune stimulation cytokines (e.g., IFN-*γ* and GM-CSF) reverse monocyte paralysis after burns, restoring antigen presentation and correcting immune defects (364, 365). Immune nutrients like glutamine and omega-3 fatty acids restore inflammation-immune balance by maintaining intestinal barrier integrity and promoting anti-inflammatory mediators (366). Cellular immune therapy, including mesenchymal stem cells, regulates immune cell polarization and promotes tissue repair via paracrine mechanisms, yet clinical trials confirm safety and effectiveness locally, though preparation protocols remain inconsistent and standardization insufficient (367, 368). Adoptive infusion of regulatory T cells can inhibit uncontrolled inflammation, though it remains in pre-clinical research with significant clinical potential (369). Comprehensive auxiliary interventions can further improve systemic inflammation. Blood purification techniques like CRRT and cytokine adsorption may reduce excess inflammatory mediators, but large-scale randomized controlled trials are lacking (370). Probiotics and prebiotics protect intestinal barrier function and ameliorate immune dysregulation (371). Emerging technologies are advancing precise immune therapy for burns. Epigenetic regulators can balance inflammation and immune suppression (372). Nano drug delivery systems enable targeted drug release, enhancing efficacy while reducing side effects (373, 374). Biomarkers such as immune cell subsets allow differentiated interventions, with intensive anti-inflammatory therapy for severe inflammation and targeted immunostimulatory therapy for immunosuppression. This strategy offers new insights for future precision burn treatment (375).

In general, anti-inflammatory and immune regulation strategies each have advantages and can be combined, addressing immune inflammation-mediated organ injury after burn and providing a solid basis for clinical treatment.

#### 4.2.4 Extracorporeal blood purification and immune modulation

When applying extracorporeal blood purification techniques in clinical practice, it is essential to clearly define the scope of application and treatment goals for each technique. CRRT is a traditional form of renal replacement therapy used to correct volume overload, electrolyte imbalances, and uremia caused by burn-related acute kidney injury. This method stabilizes fluid balance and removes metabolic waste; notably, CRRT serves solely as a means of organ support and does not possess specific immunomodulatory value (320). However, the optimal timing for initiating CRRT remains controversial. Although some reports indicate that renal replacement therapy improves survival rates 54 in patients requiring vasopressors and in shock, However, the statistical power of these analyses was insufficient; large-scale randomized controlled trials are still needed to assess whether this treatment approach offers survival benefits for any other burn subgroups (376).

High-volume hemofiltration (HVHF) theoretically suppresses the systemic inflammatory response by removing inflammatory mediators from the circulation; however, existing burn cohort data have not yet demonstrated its ability to reduce mortality or sustainably lower levels of inflammatory markers. Previous studies have shown that although HVHF can reduce levels of inflammatory and endothelial damage markers in the early phase (within 6 hours), its effects are transient, followed by a rebound in mediator levels and the reappearance of cellular damage within 48 hours, indicating that its biological effects are time-limited and do not provide sustained immunomodulatory benefits (377). There is no clear evidence of a survival benefit from HVHF. Therefore, it should not be regarded as a reliable routine immunomodulatory regimen.

Adsorption-based therapies, including coupled plasma filtration and adsorption (CPFA) and direct blood adsorption (HA), represent more selective *in vitro* clearance strategies. For burn patients with concurrent septic shock and acute kidney injury, these techniques can improve hemodynamic parameters, however, their potential survival benefits have only been observed in small-sample, retrospective, uncontrolled studies and can serve only as a basis for hypothesis generation, without yet confirming their definitive efficacy. Innovative studies have found that novel, highly biocompatible adsorbent materials can modulate burn-induced inflammation and immune dysregulation (378), but due to the nonspecific removal characteristic of most HA techniques, it has also been observed that adsorption therapy inadvertently removes anti-infective drugs in sepsis. Adsorption clearance is inconsistent, and there are currently no guidelines providing dosage recommendations for medications during HA. Research confirming its safety, efficacy, and practicality for use in cases of poisoning remains insufficient (379). Furthermore, a recent study discovered quorum-sensing molecules in the plasma of burn patients suffering from septic shock, along with antibiotics, and demonstrated that *in vitro* adsorption can remove them. However, this does not equate to the ability of adsorption therapy to effectively remove these molecules *in vivo*, and there is currently no evidence that this translates into improved infection control or survival benefits (380).

A recent study found that, for burn patients with both acute kidney injury and septic shock, the combination of plasma filtration and adsorption significantly reduced mortality compared to conventional CRRT (381). Another observational study of a similar cohort of burn patients with septic shock also confirmed that the combined use of adsorption therapy and continuous renal replacement therapy CRRT yields better treatment outcomes (382). Although the results of these studies show improvements in both hemodynamic parameters and survival rates, current clinical evidence does not yet support the use of extracorporeal blood purification as a definitive anti-inflammatory treatment (378). This is primarily due to limited sample sizes and the lack of adequately powered randomized trials. These studies currently remain hypothesis-generating in nature. It is worth noting that recent commentary has explicitly pointed out that, compared with patients with ordinary sepsis, adsorption therapy offers significantly greater clinical value in burn patients who have concurrent septic shock and require continuous renal replacement therapy (383). This suggests that future multicenter clinical trials should pay close attention to the selection of study participants and the timing of intervention in order to better explore the therapeutic efficacy and clinical impact of adsorption therapy (384). Assessing immunomodulatory effects requires burn-specific data to distinguish between early- and late-onset acute kidney injury. For severe burn-associated multiple organ dysfunction, the combined use of ECMO and CRRT represents an important direction for future research (344). Therefore, until these issues are resolved, the use of extracorporeal therapies for burn-related immunomodulation should remain limited to the research phase.

### 4.3 Metabolic regulation and pharmacological intervention

#### 4.3.1 Metabolic regulation and personalized nutritional support

Severe burns can cause persistent metabolic disorders that interact with multi-organ injuries, forming a vicious cycle. Metabolic regulation and personalized nutrition support are key interventions to interrupt this process, aiming to correct metabolic imbalance, reduce inflammation, and protect organ function. A comprehensive treatment system now includes accurate nutrition supply, targeted immune regulation, and drug metabolism intervention (385). Nutritional support requires precise assessment for proper macronutrient and micronutrient proportions. Resting energy expenditure is measured by indirect calorimetry, with caloric provision set at 100%–130% of the target to prevent organ overload (386). Daily supplementation of 1.5–2.0g/kg of high-quality protein (387), prioritizing branched chain amino acid (BCAA) preparations, helps counteract muscle catabolism and supports organ function and wound healing. Patients with acute kidney injury require careful adjustment of nitrogen intake to maintain homeostasis (384).

Targeted immune nutrients enhance organ protection. Glutamine stabilizes the intestinal barrier, reduces endotoxin shift, and lessens secondary liver and lung injury (388). Glutamate-3 polyunsaturated fatty acid reshapes inflammatory mediators and reduces systemic inflammation. Arginine should be used cautiously in early burn stages to avoid hemodynamic instability (345). Zinc, selenium, vitamin C, and vitamin E restore antioxidant enzyme activity, reduce oxidative stress damage, and improve immune function (345, 389). Intestinal microecology regulation improves systemic metabolism. Prebiotics, probiotics, and soluble fiber reshape gut flora, maintain mucosal integrity (345), and reduce organ damage. Emerging technologies like vagus nerve stimulation offer novel strategies for intestinal barrier protection (390).

Individualized dynamic adjustment is key for intervention efficacy, requiring real-time monitoring of cardiac, hepatic, renal, and pulmonary function and dynamic optimization of nutritional regimens. Patients with hepatic dysfunction should prioritize medium- and long-chain triglyceride emulsions or omega-3 fatty acid-rich lipid formulations (391). Those with pulmonary injury may adopt a permissive low-calorie strategy (392). Initiate early enteral nutrition within 24 hours, and combine it with parenteral support when intake remains inadequate while taking precautions against catheter-related infections (393, 394). Assess therapy efficacy by measuring levels of short-half-life proteins, monitor glucose, lipid, and electrolyte levels, and prevent metabolic complications such as refeeding syndrome. Accurate nutrition and metabolic intervention can break the cycle of hypermetabolism and organ injury, facilitating severe burn treatment (392).

#### 4.3.2 Clinical pharmacology regulation strategies

Previous studies have confirmed that burn patients remain in a state of persistent inflammation and hypermetabolism for up to three years post-injury. As metabolic hyperactivity intensifies, resting energy expenditure increases significantly, and levels of cortisol, catecholamines, cytokines, and acute-phase proteins rise markedly, accompanied by physiological alterations including insulin resistance. This predisposes patients to long-term organ damage and a decline in physical function (18). Consequently, clinical pharmacological interventions targeting long-term hypermetabolic states and organ dysfunction following burns have gradually shifted from empirical supportive therapy to metabolically targeted precision regulation. In the future, multicenter long-term follow-up data should be utilized to determine the optimal timing, dosage, and combination regimens for different drugs in organ protection (111), with the aim of improving patient outcomes. See **Table 2** for details.

**Table 2:** Clinical use of drugs for long-term organ damage in burn patients.

Drug name	Drug categories	Mechanism of action	Effects	Clinical limitations	References
Propranolol	*β*-blockers (sympatholytic agents)	Antagonizes catecholamine-mediated sympathetic overactivity	Lower heart rate, reduce myocardial oxygen use, prevent damage, inhibit excess protein/fat breakdown, improve protein balance, ease hypermetabolism.	Heart rate and blood pressure must be monitored, and medication dosages must be carefully controlled	(395)
Oxandrolone	Androgen receptor agonist (anabolic androgenic steroids).	Stimulates androgen receptors to promote transcriptional regulation of protein synthesis.	Enhances muscle protein synthesis, corrects nitrogen balance, stimulates hepatic protein synthesis, shortens hospital stay, and reduces wound infection risk.	Randomized controlled trials exist, but oxandrolone's long-term effects on organ fibrosis remain unclear, requiring further large-scale studies.	(125)
Insulin	Antidiabetic and metabolic regulators	Binds to the insulin receptor, activating PI3K/Akt signaling, increasing glucose uptake, promoting protein and lipid synthesis, and inhibiting protein degradation and hepatic gluconeogenesis.	Proteolysis inhibition and post-burn hyperglycemia restoration	Intensified insulin therapy requires close monitoring of blood glucose levels to prevent the risk of hypoglycemia	(396)
Metformin	Antidiabetic and metabolic regulators	Inhibits mitochondrial complex I, raising AMP/ATP to activate AMPK, blocks hepatic gluconeogenesis, and modulates lipid metabolism, oxidative stress, and inflammation.	Reduces hepatic lipid buildup and mitochondrial stress, protects liver, improves cardiac metabolism, and has anti-inflammatory effects beyond glucose control.	The organ-protective efficacy remains to be validated in large-scale prospective clinical trials.	(397)
Recombinant human growth hormone, IGF-1	Growth endocrine axis agents	Binds growth hormone receptor (GHR) to activate. Stimulates protein anabolism, and promotes cellular proliferation.	Involved in the long-term repair of tissue damaged by burns.	May cause hyperglycemia and fluid retention; its use in severely septic or critically ill burn patients is linked to increased mortality, making routine clinical use controversial.	(398)
Statins	Antifibrotic and vascular protective drugs	Inhibits HMG-CoA reductase, the key enzyme in cholesterol synthesis, and has anti-inflammatory, antioxidant, and pro-angiogenic effects that aid tissue repair.	Promotes wound healing via anti-inflammatory, antioxidant, and pro-angiogenic effects, and may modulate CYP19A1 expression.	Long-term organ protection efficacy requires confirmation from large-scale trials.	(399)
Angiotensin-converting enzyme inhibitors (ACEIs)	Antifibrotic and vascular protective drugs	Competitively inhibits ACE, blocking angiotensin II conversion, thereby reducing vasoconstriction, aldosterone secretion, and pro-fibrotic (TGF-*β*/Smad) and pro-inflammatory pathways.	Reduces vascular endothelial damage and alleviates myocardial fibrosis	Despite strong evidence in cardiovascular diseases, ACEIs' efficacy for long-term organ protection in burn patients remains uncertain, requiring further large-scale trials.	(400)

### 4.4 Applications of regenerative medicine and cell therapy in organ repair

Recent studies have shown that the application of regenerative medicine and cell therapy in organ repair has attracted significant attention. Notably, stem cells exhibit exceptional anti-inflammatory, immunomodulatory, and tissue-regenerative capabilities. Exosomes derived from mesenchymal stem cells demonstrate low immunogenicity, high biocompatibility, and potent paracrine regulatory functions. Currently, these technologies represent cutting-edge research avenues for the regeneration and repair of multi-organ injuries following burns, effectively addressing the limitations of traditional cell therapy (401). Consequently, they have emerged as a pivotal therapeutic strategy for managing multi-organ injuries following severe burns (402) (**Table 3**).

**Table 3:** Applications of regenerative medicine and cell therapy in organ repair.

Target organs	Key signaling pathways	Mechanism of action and effects	Types of clinical evidence	Clinical limitations
Heart	Bax/Bcl-2 apoptosis pathway NLRP3 inflammasome	MSCs homing to cardiac microvasculature upregulate IGF-1 via paracrine signaling, modulating the Bax/Bcl-2 pathway to inhibit cardiomyocyte apoptosis and improve post-burn myocardial depression. S100A9-positive exosomes from burn patients activate NLRP3 inflammasome and induce pyroptosis. Stem cell-derived exosomes counteract this by delivering protective nucleic acids and proteins, exerting cardioprotection.	Animal model (rat model) Human tissue or biomarker data	Variability in stem cell sources, lack of standardized protocols (timing/dosage) (403). Burn models differ from clinical patients, have inconsistent preparation methods, and lack reproducibility (404).
Liver	Regulation of the local immune microenvironment	Modulates the local immune microenvironment, reduces the release of pro-inflammatory factors, transports antioxidant mRNA, protects hepatocytes, and counteracts ischemic and toxic damage. Specific molecular markers can reflect the extent of damage in real time.	Animal model (mouse model)	Research on burns is limited, worsened by gaps between experimental models and clinical cases, and a lack of standardized protocols (405).
Lungs	SDF-1/CXCR4 signaling, PI3K/AKT, Nrf2/HO-1, p38 MAPK	Mesenchymal stem cells (MSCs) or their exosomes promote M2 macrophage polarization, enhance alveolar barrier integrity, reduce inflammation, and alleviate pulmonary edema. However, burns divert stem cells from the lungs via SDF-1/CXCR4 signaling, weakening protection. MSC exosomal miR-451 inhibits MIF, activates PI3K/AKT and Nrf2/HO-1 pathways, and inhibits ferroptosis, countering inflammation and oxidative stress. Conversely, S100A9-containing exosomes from burn serum activate p38 MAPK, disrupt tight junctions (e.g., occludin, ZO-1), and worsen pulmonary edema.	Animal model (rat model) Animal model (mouse model)	SDF-1 signaling at the wound site diverts stem cell migration, impairs lung repair, and compromises targeted therapy stability (406, 407). Lack of standardized preparation, unclear *in vivo* pharmacokinetics, and unestablished long-term safety (408, 409).
Kidney	Bax/Bcl-2 apoptosis pathway Local immune microenvironment regulation	MSCs home to the kidney, protect tubular cells via paracrine IGF-1 and Bax/Bcl-2 modulation, suppressing inflammation, optimizing repair, and improving renal function. MSC exosomes modulate immune responses, reduce pro-inflammatory cytokines, and deliver antioxidant mRNAs to protect against ischemic and toxic damage.	Animal model (rat model) Animal model (mouse model)	Research on burn-specific renal repair lacks uniform intervention standards and multicenter clinical validation data (410). Studies on burns are limited, models differ from clinical patients, and preparation methods are not standardized (411).
Multiorgan (Common Mechanism)	Paracrine signaling, immune remodeling, mitochondrial translocation.	MSCs and their derivatives protect multiple organs via three interconnected mechanisms: (1) Paracrine signaling: secreting IGF-1, TGF-β, and exosomes with proteins/miRNAs to suppress inflammation, inhibit apoptosis, and promote angiogenesis. (2) Immune remodeling: balancing immunity, promoting M2 macrophages, and reducing pro-inflammatory T cells to halt organ failure. (3) Mitochondrial transfer: delivering healthy mitochondria to cells with impaired metabolism via tunneling nanotubes or vesicles, improving energy production and reducing oxidative stress.	Animal model (rat and mouse model)	Induced pluripotent stem cells carry oncogenic risk (412). Allogeneic stem cell immunological safety is unconfirmed (413). Standardized preparation, quality control, and large-scale RCT validation are lacking (413).

### 4.5 Expanding role of tissue engineering and biological materials

Tissue Engineering and Regenerative Medicine provide an innovative technical framework for the complete repair of organ parenchyma damage and function loss after burns. (**Table 4**).

**Table 4:** The role of tissue engineering and biomaterials.

Target organs	Biomaterials	Mechanisms of core damage repair	Repair strategies and effects	Types of evidence	Clinical limitations	References
Lungs	Decellularized matrix hydrogel, hyaluronic acid.	Alveolar structure and reconstruction of the qi-blood barrier.	Stem cells delivered via airways with hyaluronic acid hydrogels improve cell retention and reduce lung inflammation. Drug-loaded scaffolds reduce pulmonary fibrosis. Implantable artificial lung modules can temporarily perform gas exchange.	Rat model	Lack of sufficient evidence regarding the safety of biomaterial degradation and long-term stability following host integration. Immune rejection caused by heterogeneity of patient injuries.	(414)
Kidney	Decellularized renal matrix and exosome hybrid hydrogel.	Renal tubule unit regeneration	Renal tubular epithelial cells are seeded onto scaffolds to create biomimetic tubules, a key component of bioartificial kidney devices when combined with filtration systems. Decellularized matrices attract endogenous stem cells and offer tissue-specific biochemical cues. Exosome-loaded hydrogels release reparative factors to reduce renal tubular apoptosis and inflammation.	Animal model (rat and mouse model)	Technology for large-scale, standardized mass production is not yet mature. It is difficult to produce complex biomimetic tissues in bulk.	(415)
Liver	Alginate microspheres, vascularized porous scaffolds, decellularized liver matrix, immunomodulatory biomaterials.	Reconstruction of liver lobular structure, temporary hepatic function replacement, and immunomodulation for regenerative microenvironment remodeling.	Hepatocytes on alginate microspheres offer temporary hepatic support as a bridge-to-recovery, while those on porous scaffolds aim for long-term liver substitutes. Vascularized organoids are created using the extracellular matrix (ECM) of decellularized liver tissue and are cultured in three dimensions within a microfluidic system. Immunomodulatory biomaterials remodel the regenerative environment and reduce inflammation.	Animal model (rat and mouse model)	Allogeneic materials and cells often trigger rejection reactions. There is insufficient evidence of long-term stability following integration into the host.	(416, 417)
General	Natural materials (e.g., decellularized ECM scaffolds, polysaccharides (hyaluronic acid, chitosan, alginate), and proteins (collagen, gelatin, fibronectin)) and synthetic polymers (e.g., PLGA, PEG, PCL).	Biomimetic scaffolds enable structural reconstruction and functional restoration by providing 3D support, delivering bioactive factors, and regulating cellular behaviors.	Precise, time-controlled delivery of bioactive factors (such as the sequential release of VEGF and HGF) via biomaterial carriers. Enzyme-responsive delivery systems enable on-demand release. Delivery of nucleic acid therapeutics such as siRNA and miRNA via cationic polymers, lipid nanoparticles, or scaffold-based loading systems for local gene intervention.	Animal model (rat and mouse model)	Challenges in clinical translation: immature large-scale production technologies, safety and long-term stability issues related to the degradation of biomaterials, and immunocompatibility issues with heterologous materials and cells.	(418–421)
